# Specific nanoprobe design for MRI: Targeting laminin in the blood-brain barrier to follow alteration due to neuroinflammation

**DOI:** 10.1371/journal.pone.0302031

**Published:** 2024-04-11

**Authors:** Juan F. Zapata-Acevedo, Mónica Losada-Barragán, Johann F. Osma, Juan C. Cruz, Andreas Reiber, Klaus G. Petry, Amael Caillard, Audrey Sauldubois, Daniel Llamosa Pérez, Aníbal José Morillo Zárate, Sonia Bermúdez Muñoz, Agustín Daza Moreno, Rafaela V. Silva, Carmen Infante-Duarte, William Chamorro-Coral, Rodrigo E. González-Reyes, Karina Vargas-Sánchez

**Affiliations:** 1 Grupo de Investigación en Neurociencias (NeURos), Centro de Neurociencia Neurovitae-UR, Instituto de Medicina Traslacional (IMT), Escuela de Medicina y Ciencias de la Salud, Universidad del Rosario, Bogotá, Colombia; 2 Grupo de Biología Celular y Funcional e Ingeniería de Biomoleculas, Departamento de Biología, Universidad Antonio Nariño, Bogotá, Colombia; 3 Department of Electrical and Electronic Engineering, Universidad de los Andes, Bogotá, Colombia; 4 Department of Biomedical Engineering, Universidad de los Andes, Bogotá, Colombia; 5 Chemistry Department, Grupo La Quimica en la interfase inorgánica-orgánica QUINORG, Universidad de los Andes, Bogotá, Colombia; 6 CNRS UMR 5536 Centre de Resonance Magnétique des Systemes Biologiques and INSERM U1049 Neuroinflammation, University of Bordeaux, Bordeaux, France; 7 GREMI, UMR7344, University of Orleans, Orleans, France; 8 Facultad de Ciencias, Grupo Investigación fundamental y aplicada en Materiales, Universidad Antonio Nariño, Bogotá, Colombia; 9 Médico Radiólogo Institucional, Fundación Santa Fé de Bogotá, Bogotá, Colombia; 10 Oficial de Protección Radiológica, Fundación Santa Fé de Bogotá, Bogotá, Colombia; 11 Experimental and Clinical Research Center, a Cooperation between the Max Delbrück Center for Molecular Medicine in the Helmholtz Association and Charité—Universitätsmedizin Berlin, Berlin, Germany; 12 Laboratorio de Neurofisiología Celular, Grupo de Neurociencia Traslacional, Facultad de Medicina, Universidad de los Andes, Bogotá, Colombia; Cedars-Sinai Medical Center, UNITED STATES

## Abstract

Chronic neuroinflammation is characterized by increased blood-brain barrier (BBB) permeability, leading to molecular changes in the central nervous system that can be explored with biomarkers of active neuroinflammatory processes. Magnetic resonance imaging (MRI) has contributed to detecting lesions and permeability of the BBB. Ultra-small superparamagnetic particles of iron oxide (USPIO) are used as contrast agents to improve MRI observations. Therefore, we validate the interaction of peptide-88 with laminin, vectorized on USPIO, to explore BBB molecular alterations occurring during neuroinflammation as a potential tool for use in MRI. The specific labeling of NPS-P88 was verified in endothelial cells (hCMEC/D3) and astrocytes (T98G) under inflammation induced by interleukin 1β (IL-1β) for 3 and 24 hours. IL-1β for 3 hours in hCMEC/D3 cells increased their co-localization with NPS-P88, compared with controls. At 24 hours, no significant differences were observed between groups. In T98G cells, NPS-P88 showed similar nonspecific labeling among treatments. These results indicate that NPS-P88 has a higher affinity towards brain endothelial cells than astrocytes under inflammation. This affinity decreases over time with reduced laminin expression. *In vivo* results suggest that following a 30-minute post-injection, there is an increased presence of NPS-P88 in the blood and brain, diminishing over time. Lastly, EAE animals displayed a significant accumulation of NPS-P88 in MRI, primarily in the cortex, attributed to inflammation and disruption of the BBB. Altogether, these results revealed NPS-P88 as a biomarker to evaluate changes in the BBB due to neuroinflammation by MRI in biological models targeting laminin.

## 1. Introduction

The blood-brain barrier (BBB), functionally composed of brain endothelial cells, pericytes and astrocytes, is fundamental for the homeostasis of the central nervous system (CNS). It controls and regulates the passage of ions, molecules and cells into the brain and spinal cord [1]. In neurodegenerative disease, chronic neuroinflammation is characterized by the constant presence of damaging inflammatory stimuli that increase the permeability of the BBB, mainly due to alterations in basal lamina proteins, membrane receptors, and endothelial cell tight junctions [2]. As the BBB is crucial to preserve the functional integrity of the CNS, it is necessary to develop and validate new molecular techniques and tools for identifying and monitoring changes involved in the maintenance, structure, and function of the BBB, including molecular changes such as enzyme activation or changes in protein abundance.

Advanced imaging techniques, such as magnetic resonance imaging (MRI), have contributed to detecting lesions and permeability of the BBB [3]. Its high sensitivity to detect inflammatory and neurodegenerative processes in the brain has made it a powerful tool to monitor the progression of diseases such as multiple sclerosis (MS) and to help differentiate a healthy from a compromised BBB in different brain regions [4]. In addition, contrast agents have been confirmed to improve MRI observations in the brain [5]. The most used contrast agents are based on Gd-chelates, as they modify the longitudinal relaxation time (T_1_). These compounds have been classified as positive contrast agents because they show a hyperintense signal. However, their use may be risky in some cases as they can accumulate in certain organs and tissues [6, 7]. Ultrasmall superparamagnetic particles of iron oxide (USPIO) represent an alternative to Gd-chelates and a promising contrast agent for MRI observations [8, 9]. In MS, USPIO can increase the contrast and improve distinct imaging interpretation of cellular phagocytic (macrophage/microglia) inflammatory lesion activity at the BBB and within brain parenchyma compared to Gd [8–13]. USPIO are nanoparticles (NPS) with diameters below 50 nm composed of magnetite (Fe_3_O_4_) and/or maghemite (γ-(Fe_3_O_4_), and show a superparamagnetic behavior, resulting from their small size and crystalline nature, which increases their magnetic susceptibility. USPIO generate a hypointense signal as they modify the transversal relaxation time (T_2_) and are used mainly for T2-weighted MRI sequences [14–16].

However, the development of imaging tools with high specificity to visualize cellular changes and molecular and functional alterations in the BBB is still needed. There is strong interest in the vectorization of NPS with selected peptides that exclusively target a specific receptor to track at different stages, the progression of neurodegenerative diseases. For instance, NPS carrying the VHSPNKK peptide target exclusively the vascular cell adhesion molecule-1 (VCAM-1)-expressing endothelial cells and have been used for *in vivo* MRI analysis [17–19]. In Alzheimer’s disease (AD), USPIO vectorized with a heptapeptide are used due to their ability to target amyloid plaques [20].

Our previous research allowed the identification of peptide biomarkers against the BBB under neuroinflammatory conditions, using phage display screening in rats with autoimmune encephalomyelitis (EAE) [21]. Among the panel of EAE-specific labeling phages explored, the phage clone 88 efficiently and specifically bound to blood vessels of the spinal cord and brain tissue of EAE-developing rats but showed no labeling in control animals.

To elucidate the molecular target of the peptide, a crosslinking was made with phage clone 88, which carries the peptide 88 (P88), and human cortical microvessel endothelial cells (hCMEC/D3 cell line) under inflammatory conditions [22]. P88 displayed a high affinity for target proteins in hCMEC/D3 cells stimulated with interleukin-1β (IL-1β), whereas no specific binding was observed with non-stimulated cells. P88 presented significant binding to the laminin subunit β1, but showed no binding to fibronectin, flotillin-1, or laminin subunit α5. Thus, our previous work revealed that P88 is highly selective towards the laminin subunit β1 under inflammatory conditions [22].

Laminins are glycoproteins that constitute the major non-collagenous components of basement membranes (BMs) [23]. Laminins are composed of α, β, and γ subunits [24]. The α subunit, the largest of the three subunits, interacts with cell receptors such as integrins and dystroglycans. In contrast, the β and γ subunits interact with extracellular matrix (ECM) molecules such as collagen and nidogen [25]. Several reports suggest the involvement of laminin in regulating the BBB function and neuroinflammation [26–32]. Although endothelial cells, pericytes, and astrocytes can produce laminin, each one expresses different isoforms of the protein [29, 33, 34]. For example, the major isoform expressed in normal brain vascular endothelium is laminin 421 (α4-β2-γ1) [35]. Moreover, specific isoforms of laminin, such as the laminin-511 (α5-β1-γ1), has been reported to improve the integrity of the BBB by stabilizing tight junctions, increasing cell–cell adhesion strength, and reducing leukocyte extravasation under neuroinflammatory conditions [36]. In addition, higher expression of laminin-511 has been shown to inhibit T lymphocyte migration, preventing interactions between lymphocytes, α6β1 integrin, and laminin-411 [37]. Furthermore, the expression of laminin changes depending on the type of neuroinflammation. Under acute neuroinflammation, the expression of laminin decreases, but during chronic neuroinflammation, the expression of laminin increases over time [38].

Laminin can be proposed as a potential biomarker for neurological diseases with a neuroinflammatory component. The development and validation of nanoprobes directed at laminin and based on USPIO as contrast agents is of special interest due to their MRI applications. These nanoprobes may allow the visualization of specific biological alterations in the BBB at the cellular and molecular level and monitor the progression of the disease. This work aims to validate a nanoprobe designed as a USPIO MRI contrast agent vectorized with the selected P88, which targets laminin under neuroinflammatory conditions.

## 2. Results

### 2.1 Synthesis and characterization of iron oxide-based nanoprobes

Different iron oxide crystalline phases can be obtained depending on the Fe oxidation state, and these include wüstite (FeO), hematite (α‐Fe_2_O_3_), maghemite (γ‐Fe_2_O_3_) and magnetite (Fe_3_O_4_). Raman spectroscopy was performed on bare iron oxide NPS to determine the presence of different crystalline phases based on their vibration modes. S1A Fig shows the Raman spectrum of bare iron oxide NPS. The spectrum presents two intense peaks at 664 and 709 cm^-1^, confirming the presence of magnetite (full diamond symbol) and maghemite (open triangle symbol) phases respectively. However, maghemite could have a contribution to the signal at 664 cm^-1^. Peaks at 377 cm^-1^ and 496 cm^-1^ could be assigned to the maghemite phase, while the peaks at 332 cm^-1^ and 562 cm^-1^ correspond to the magnetite phase [39–43]. Magnetite and maghemite are suitable phases to be used as contrast agents in MRI due to their magnetic properties [44].

The hydrodynamic diameter (HD) of bare and silanized iron oxide NPS was measured using dynamic light scattering (DLS) (S1B Fig). The obtained particle size distribution reveals that after silanization there is a slight increase of the diameter from 134 to 156 nm, which is accompanied by a broadening of the distribution as evidenced by the polydispersity index (PDI), that indicates the dispersion degree of the NPS [45], that increases from 0.134 to 0.196 for bare and silanized NPS respectively. This HD increase concurs with previous results after the silanization of NPS [46].

Fourier transformed infrared spectroscopy (FTIR) measurements were performed to investigate changes in the surface chemistry of NPS (S1C Fig). For bare iron oxide NPS, the FTIR spectrum shows an intense signal at 576 cm^-1^ that can be assigned to the Fe-O stretching vibration of the magnetite phase, while the shoulder observed at 632 cm^-1^ corresponds to the Fe-O stretching vibration of the maghemite phase [47–50]. This result confirms that bare NPS are a mixture of magnetite and maghemite phases as observed with the Raman results. After the silanization process, the FTIR spectrum shows additional bands at 1132 cm^-1^ and 1082 cm^-1^, corresponding to Si–O–Si asymmetric stretching vibration modes [51–54]. The vibrational frequency located at 950 cm^−1^ can be assigned to the Si–OH bond tension and its intensity is related to the abundance of–OH groups on the surface of the NPS [55]. Moreover, bands in the 2891 cm^-1^ and 2980 cm^-1^ range correspond to the stretching of C–H bonds of the APTES [49, 52]. The bands observed at 1641 cm^−1^ correspond to the bending vibration of the free NH_2_ group after silanization, while bands at 1593 and 1466 cm^-1^ are assigned to the NH_2_ deformation modes of the amine groups [51].

The results confirmed the presence of free -NH_2_ groups that will be available for a posterior conjugation step. We evaluated the iron oxide NPS magnetic response by vibrating-sample magnetometer (VSM) measurements to verify superparamagnetism. S1D Fig shows: (i) the typical curve shape profile of a superparamagnetic material, (ii) a near-zero magnetic coercivity (magnetic field value when the moment is zero) and, (iii) a magnetic saturation of 43 emu/g, close to that reported on the literature for a superparamagnetic iron oxide [55].

**Fig 1:**
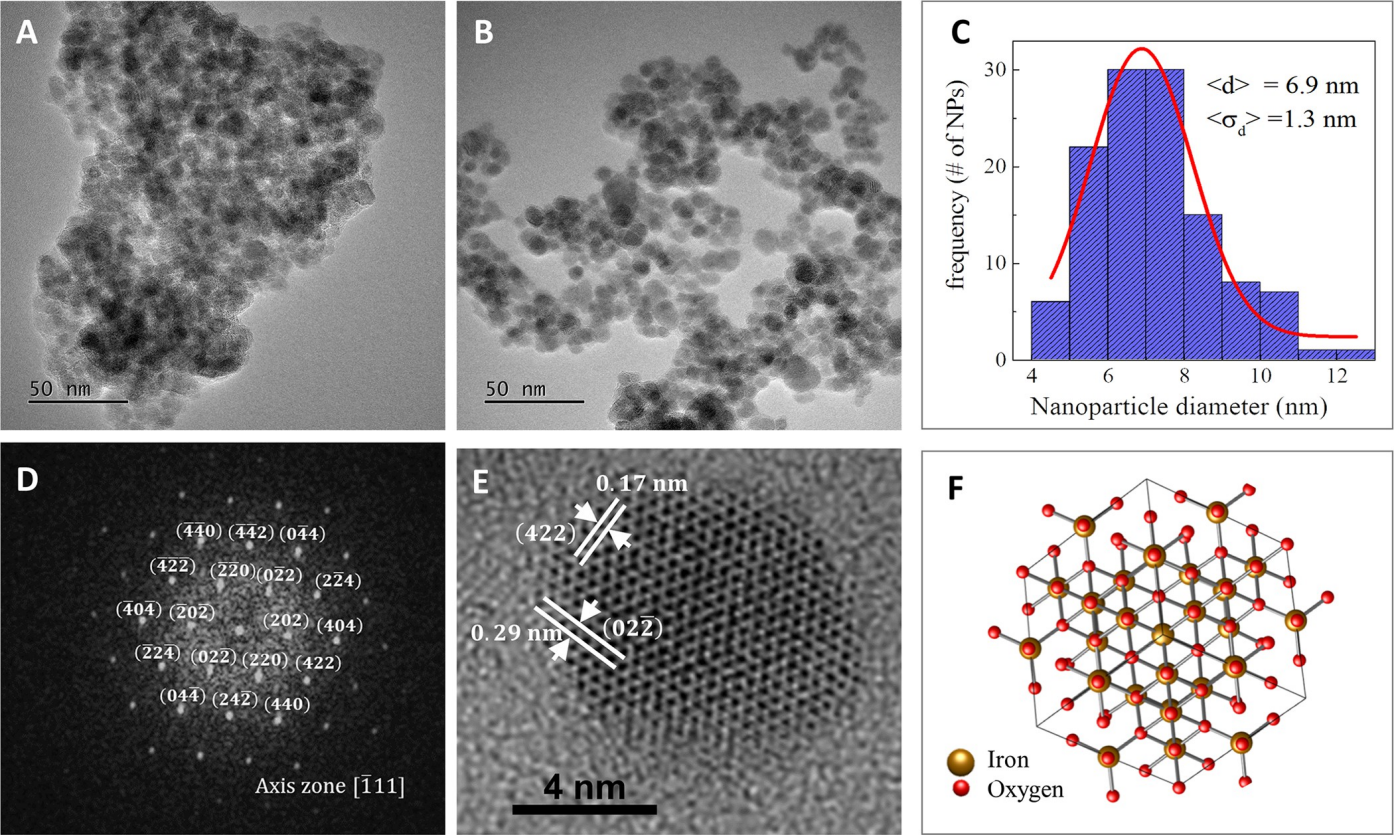
Size and crystallographic determination of NPS. TEM micrographs of bare NPS (A), silanized NPS (B) and a histogram of the NPS diameter calculated with 120 NPS (C). Indexed FFT pattern of an isolated NP (D). Each spot corresponds to a crystal plane of the magnetite phase; the axis zone was found to be [111]. HRTEM of the isolated NP (E). The FFT pattern allows the determination of the space distances of two crystallographic planes corresponding to the magnetite phase. The image in panel F represents the crystal structure of the magnetite phase in the same orientation as the NP.

Further characterization was performed to obtain information about the diameter and morphology of NPS in a dry state. Also, their crystallinity, which could be responsible for the observed magnetic properties. Fig 1 shows transmission electron microscopy (TEM) micrographs of bare (panel A) and silanized (panel B) iron oxide NPS. Silanized NPS seem to be more dispersed than the bare ones, showing that the organosilane coating is likely to decrease the NPS clustering. The histogram of Fig 1C shows that the mean diameter of silanized NPS was 6.9 ± 1.3 nm (n = 120) and shows no differences with the mean diameter of bare NPS with 7.2 ± 1.6 nm (n = 81) (histogram not shown). Importantly, the diameter found by high-resolution TEM (HRTEM) (Fig 1C) differs from the HD obtained by DLS (S1B Fig). This is because DLS measures the hydrodynamic size of NPS in aqueous suspension where interactions lead to aggregation, whereas HRTEM is conducted in a dry state. These discrepancies are explained by the differences among both techniques, as TEM measures the inorganic core of isolated NPS while DLS measures the hydrodynamic size that describes large populations of NPS in suspension [51]. Unlike TEM micrographs, the silanization process can be detected by DLS because this coating can affect the NPS behavior in suspension. This effect has been observed previously [52].

In addition, we explored the crystallinity of the NPS. We randomly chose a bare NP to perform a fast Fourier transform (FFT) of an HRTEM image to obtain reciprocal space images (Fig 1D). The FFT pattern corresponded to a NP with high crystallinity. Thus, by indexing each crystalline plane, we were able to assign this NP to the magnetite phase [55–57] (mp-19306). The axis zone of this isolated NP was found to be [111]. Fig 1E shows the HRTEM micrograph of the bare isolated NP. We identified two space distances of 0.17 nm and 0.29 nm, corresponding to the (422) and the (022) crystal planes of the magnetite phase, respectively. Fig 1F shows the crystalline structure of the magnetite phase in the same orientation as for the bare isolated NP. Silanization is a low-energy process, and it is not expected to produce a phase transition from magnetite to phases as hematite. However, we performed selected area electron diffraction (SAED) measurements to obtain general information of the crystal structure of bare and silanized NPS (see supporting information and S2 Fig). SAED patterns show the interplanar distances corresponding to the {111}, {110}, {100} and {112} families of crystallographic planes of the magnetite phase. The (222) crystallographic plane has the most intense signal (higher density of this crystallographic plane) because it is the plane with the lowest surface energy in face-centered cubic (FCC) and inverse spinel structures, and nanostructures favor crystallographic planes with lower surface energies [58, 59].

Following the previous results, we obtained NPS with an optimal size for diagnostic applications, high crystallinity and adequate crystalline phases, and a superparamagnetic response. Therefore, these iron oxide NPS have shown characteristics compatible with their potential as contrast agents for MRI.

### 2.2 USPIO as MRI contrast agent

S3 Fig shows a T2-weighted Spin Echo (SE) sequence image with a TE = 20 ms and TR = 2000 ms. The image shows that the well intensity decreases with the Fe concentration. For the T1-weighted sequence image (S3B Fig) we observed that at low TI values (i.e. 50 ms), intensity follows a similar trend as for T2 sequence when the iron concentration changes. However, for TI as high as 700 ms, an opposite behavior is observed, and the intensity increases with the iron content. Both behaviors can be explained as follows: low TI values sets the MRI measure conditions close to a T2 sequence, showing the USPIO NPS as a negative contrast (decrease of the intensity with iron presence) while at large TI values, USPIO NPS act as positive contrast agents (increase of intensity with iron presence). Both results show how the iron in the produced NPS is capable of changing the longitudinal and transversal relaxation times of the surroundings due to their high crystallinity and superparamagnetic properties making them functional as contrast agents for MRI and ready for their vectorization with the P88 and later *in-vitro* assays.

In the Supporting information, S4 Fig is shown how TE and TI variations affect the USPIO responses that could help to develop MRI contrast agents with a good signal intensity variation. Three main features were obtained from our results: a) the intensity of each well decreases as the iron concentration increases, b) for each concentration, intensity changes with TE or TR parameters, and c) the variation of the intensity is almost null for concentrations greater than 0.5 mM.

### 2.3 *In vitro* validation of the nanoprobes

**Fig 2:**
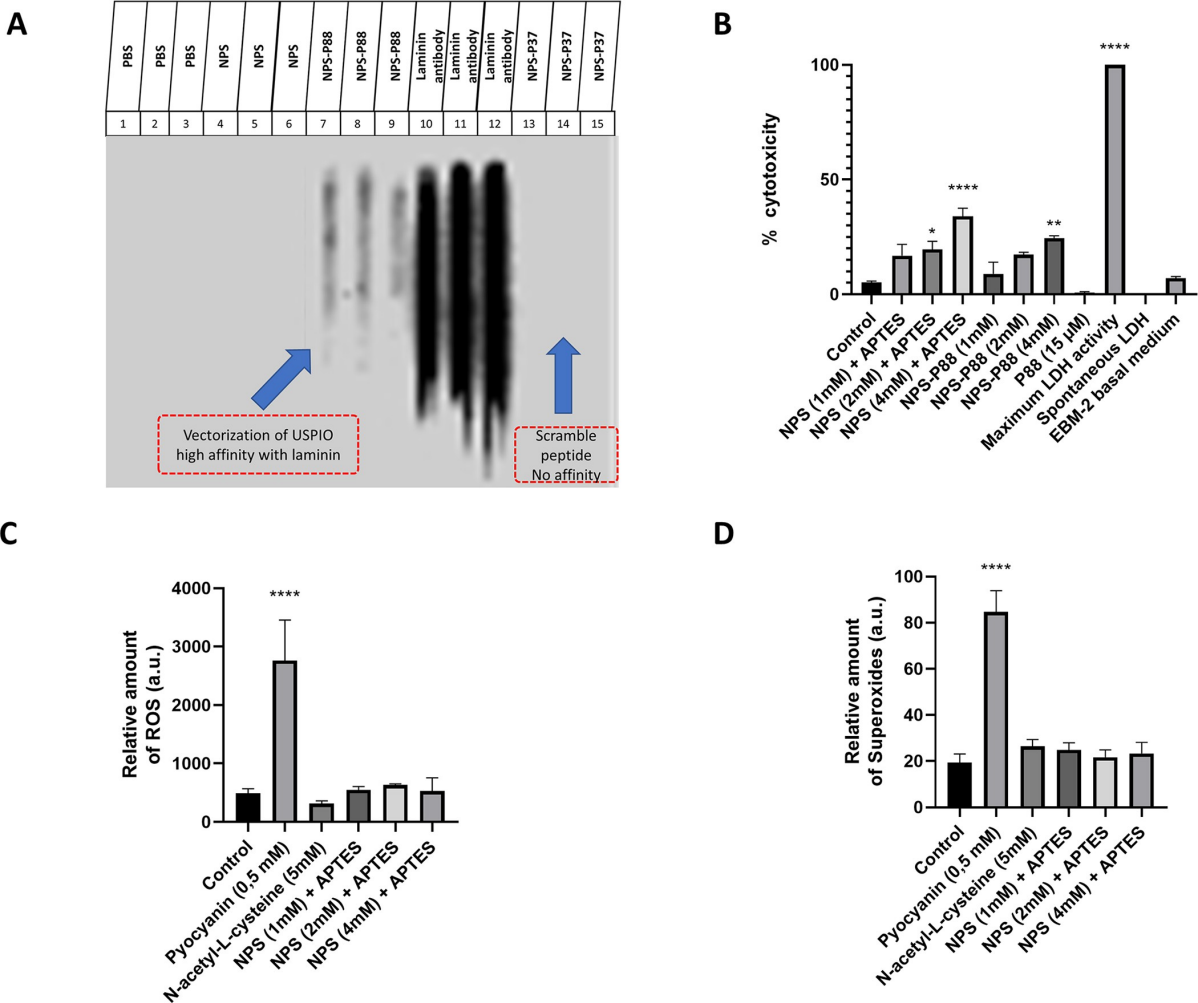
Nanoprobe laminin affinity and toxicity measurements. A) Image of the dot-blot assay nitrocellulose membrane impregnated with laminin. We demonstrated that the nanoprobe (NPS-P88) presented a high affinity to laminin, as observed in columns 7 to 9. As negative controls, we used PBS (columns 1–3), non-vectorized NPS (columns 4–6) and vectorized NPS with the scrambled peptide P37 (columns 13–15). As positive controls, we used a specific laminin antibody (columns 10–12). B) Cytotoxicity assay of NPS with or without P88 at different concentrations determined by LDH at 24 hours in hCMEC/D3 cells. Experimental groups: control (cells without treatment), cells with NPS plus APTES at different concentrations (1, 2, and 4 mM), cells with NPS vectorized with P88 at different concentrations (1, 2, and 4 mM), cells with P88 (15 μM), EBM-2 basal medium, cells with added LDH, and cells with spontaneous LDH activity. C) ROS detection at 24 hours in hCMEC/D3 cells. The relative amount of ROS was determined in untreated control cells, in cells exposed to NPS plus APTES at different concentrations (1, 2, and 4 mM), and in cells treated with pyocyanin (positive control) or N-acetyl-L-cysteine (negative control). D) Superoxide detection at 24 hours in hCMEC/D3 cells. The relative amount of superoxide was determined in untreated control cells, in cells exposed to NPS plus APTES at different concentrations (1, 2, and 4 mM), and in cells treated with pyocyanin (positive control) or N-acetyl-L-cysteine (negative control). Data are expressed as mean±SEM in arbitrary units (a.u). One-way ANOVA with Bonferroni post hoc test, * p < 0,05; ** p < 0,01; **** p < 0,0001 vs. control.

The nanoprobes were evaluated with a dot-blot assay (Fig 2A), in which a nitrocellulose membrane was impregnated with laminin β1 as the target protein. We examined the binding of the target nanoprobes functionalized with the biotinylated peptide P88 (biotin-TPMMPETSQRFK) to laminin. We used as negative controls, both the non-vectorized nanoprobes, and the vectorized nanoprobes with a scrambled peptide P37 (biotin-LPSTQPALPPNA) (NPS-P37). In addition, the laminin-specific antibody was used as a positive control. Fig 2A shows the dot-blot assay with nanoprobes vectorized to P88 (NPS-P88). The presence of a black marking reveals the interaction with laminin. Only NPS-P88 showed such labeling, demonstrating that the nanoprobe is correctly bound to P88 and the selective high affinity of P88 for laminin compared to the scrambled peptide. This assay was performed exclusively with laminin since it was the only protein with which a specific interaction with P88 was reported [22].

Cytotoxicity assays of the NPS were also performed *in vitro*, in hCMEC/D3 cells, using an LDH kit (Fig 2B). This experiment used both silanized NPS without the P88 and silanized NPS conjugated with the P88. As controls, cells without NPS or P88, cells incubated with the P88 but without NPS, and culture medium alone were used, in addition to the positive and negative controls from the LDH kit. After an incubation period of 24 hours, the silanized NPS showed no difference in cytotoxicity in hCMEC/D3 cells at a concentration of 1 mM (p = 0.28; 16.84±4.94%) when compared with control cells without NPS or P88 (5.13±0.66%). However, at 2 mM (p = 0.049; 19.59±3.537%) and at 4 mM (p< 0,0001; 34.04±3.535%), a significant increase in cytotoxicity was found when compared with control cells without NPS or P88. Likewise, the NPS conjugated with P88 at 1 mM (p>0.9999; 8.97±5.03%) and 2 mM (p = 0.215; 17.29±1.052%) showed no difference in cytotoxicity compared with control cells without NPS or P88, while a significant increase in cytotoxicity was found at a concentration of 4 mM (p = 0.0020; 24.54±1.002%). As expected, cells incubated only with P88 (p>0.9999; 0.71±0.47%), or with EBM-2 (p>0.9999; 7.00±0.78%) showed no difference when compared with control cells without NPS or P88 at 24 hours.

Once we obtained the cytotoxicity results, we decided to study whether the silanized NPS, at different concentrations, generated oxidative stress in endothelial cells. The results showed that the silanized NPS with APTES had no difference in the amount of reactive oxygen species (ROS) compared with controls at 24 hours of NPS exposure (Fig 2C). For this test, silanized NPS were used at 1, 2, and 4 mM concentrations as in the LDH assay, but without conjugating with the peptide, since here we sought to evaluate only the biocompatibility of the coated NPS. The relative amount of ROS values for the groups were: NPS (1mM) plus APTES (p>0.9999 vs. control; 547.5±56.70), NPS (2mM) plus APTES (p>0.9999 vs. control; 632.8±17.51), NPS (4mM) plus APTES (p>0.999 vs. control; 528.2±226.2), and controls (487.0±83.00). Likewise, superoxides in the cells incubated with the NPS with APTES at different concentrations showed no difference with the control cells at 24 hours of NPS exposure (Fig 2D). The relative amount of superoxides values for the groups were: NPS (1mM) plus APTES (p>0.999 vs. control; 24.83±3.146), NPS (2mM) plus APTES (p>0.999 vs. control; 21.67±3.169), NPS (4mM) plus APTES (p>0.999 vs. control; 23.25±4.854), and controls (19.33±3.801). As expected, cells treated with pyocyanin (positive control) expressed significantly higher levels of ROS and superoxides, and cells treated with N-acetyl-L-cysteine (negative control) failed to show any difference from control cells.

After verifying the specificity and conjugation of P88 to the NPS by dot-blot, and the absence of cytotoxicity or oxidative stress of NPS at a concentration of 1 mM, we decided to verify the binding of NPS-P88 in an *in vitro* model of BBB from hCMEC/D3 and T98G cells. Both cell lines were studied under inflammatory conditions induced with IL-1β (20 ng/mL). The cells were exposed to IL-1β for either 3 or 24 hours, and changes in the laminin labeling and the interaction between laminin and NPS-P88 were analyzed.

**Fig 3:**
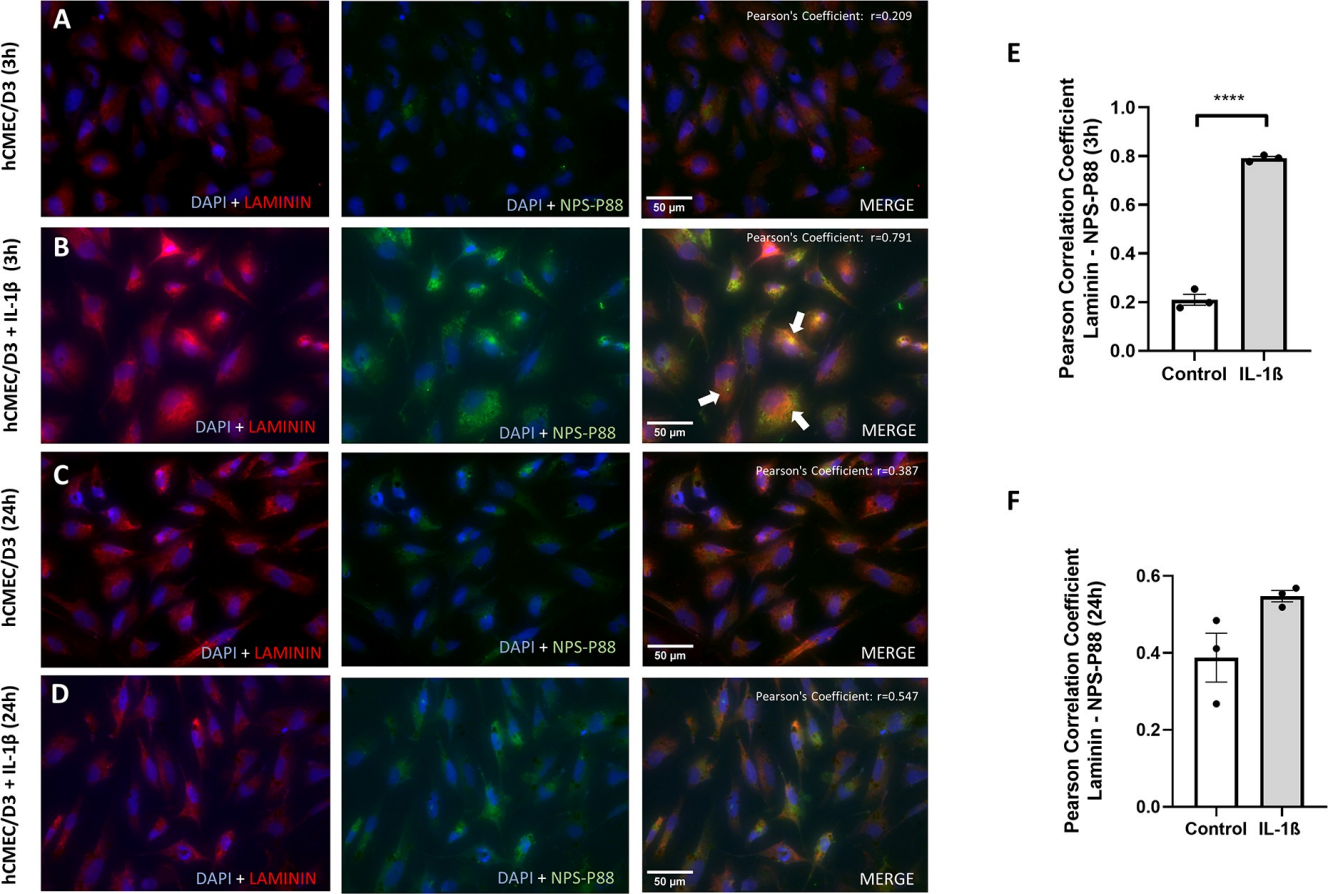
Representative images of immunofluorescence staining and colocalization analysis of nanoprobes for P88 and laminin in hCMEC/D3 cells. A) Cells without stimulation for 3 hours. B) Cells under inflammatory conditions induced by IL-1β for 3 hours. C) Cells without stimulation for 24 hours. D) Cells under inflammatory conditions induced by IL-1β for 24 hours. Blue fluorescence corresponds to DAPI, red to laminin, and green to NPS-P88. Scale bars: 50 μm. The PCC r value for the merged signal of laminin and NPS-P88 is shown in the upper right corner. Histograms representing PCC for colocalization of laminin and NPS-P88 in hCMEC/D3 are presented at 3 (E) and 24 hours (F). The mean PCC for hCMEC/D3 cells without stimulation at 3 hours was r = 0.209, while for hCMEC/D3 cells with IL-1β was r = 0.791. The mean PCC for hCMEC/D3 cells without stimulation at 24 hours was r = 0.387, while for hCMEC/D3 cells with IL-1β was r = 0.547. PCC (summarized signal) values >0.5 indicate a high probability that pixels of both channels overlay. The data are expressed with normalized mean values±SEM in arbitrary units (a.u). **** p < 0,0001 (n = 3 for panels E and F). Student t-test.

Representative immunofluorescence images of hCMEC/D3 cells treated with IL-1β for 3 and 24 hours, and stained for DAPI, laminin and NPS-P88, are shown in Fig 3. The immunofluorescence images from the NPS-P88 incubated in hCMEC/D3 showed a weak interaction without inflammatory stimulus. Likewise, laminin labeling was also low in cells without IL-1β for 3 hours (Fig 3A). However, the NPS-P88 displayed high interaction in hCMEC/D3 cells stimulated with IL-1β for 3 hours, and the laminin labeling presented a higher marking than control cells after 3 hours of the stimulus (Fig 3B). Therefore, we decided to analyze the correlation between laminin and the NPS-P88, and for this, we used the Pearson correlation coefficient (PCC). As a result, a low correlation was obtained in cells without stimulation (control), with an average of r = 0.209±0.022. In contrast, cells stimulated with IL-1β for 3 hours presented a high correlation with an average of r = 0.791±0.007 in comparison with control cells (p<0,0001) (Fig 3E).

Low labeling of NPS-P88 was found when there was no stimulation of hCMEC/D3 cells with IL-1β for 24 hours (Fig 3C), although, when stimulated with IL-1β, an apparent increase in NPS-P88 fluorescence was shown (Fig 3D). In addition, no correlation was found between laminin and NPS-P88 labeling in non-stimulated endothelial cells (r = 0.387±0.063), and a moderate correlation was found between laminin and NPS-P88 labeling when cells were stimulated with IL-1β for 24 hours (r = 0.547±0.014). However, no significant difference was obtained between unstimulated and stimulated groups (Fig 3F). Therefore, correlation values of stimulated cells are much lower at 24 hours than those observed at 3 hours. This finding indicates that the correlation between laminin and NPS-P88 is high and specific during acute inflammatory processes but decreases over time.

**Fig 4:**
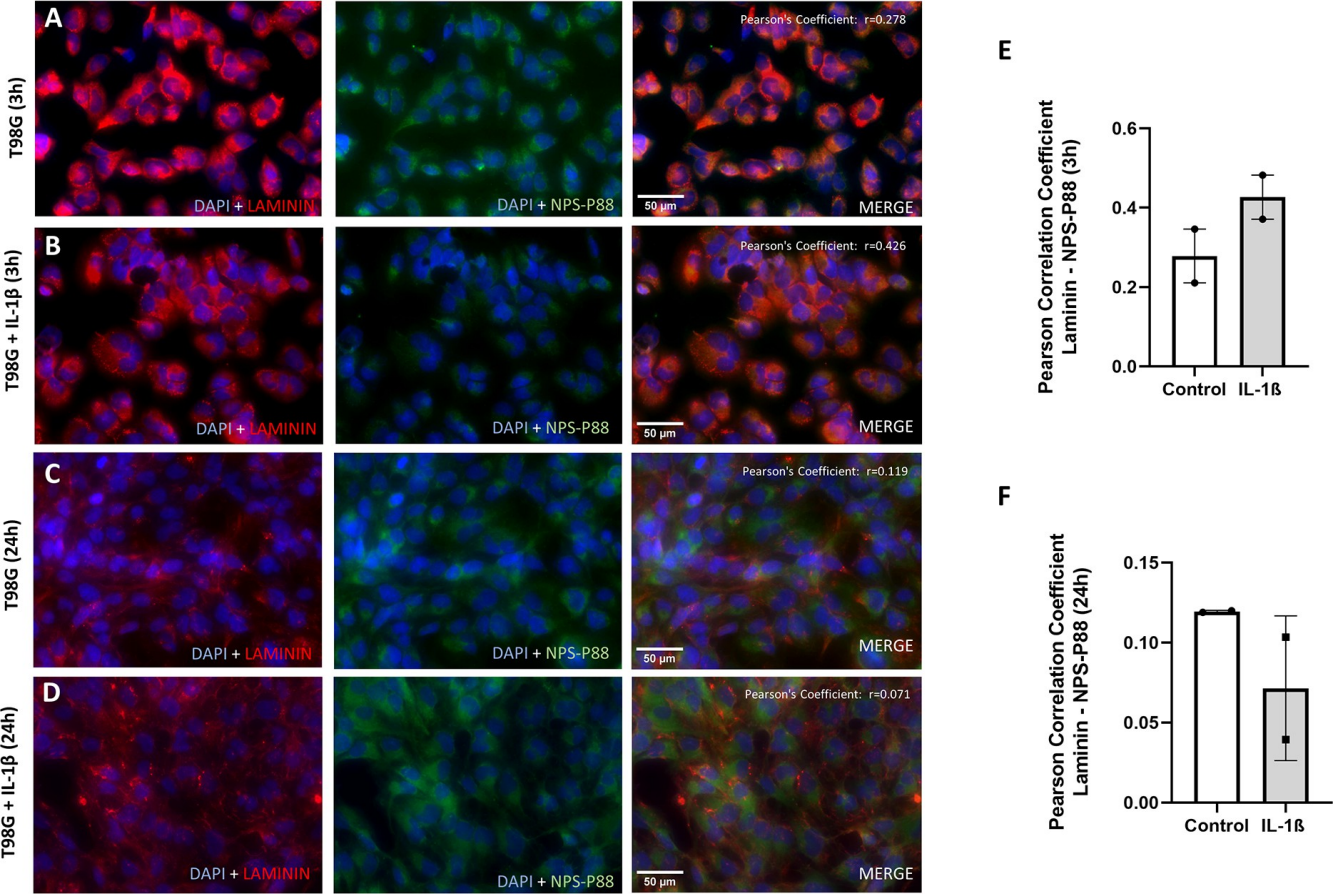
Representative images of immunofluorescence staining and colocalization analysis of nanoprobes for P88 and laminin in T98G cells. A) Cells without stimulation for 3 hours. B) Cells under inflammatory conditions induced by IL-1β for 3 hours. C) Cells without stimulation for 24 hours. D) Cells under inflammatory conditions induced by IL-1β for 24 hours. Blue fluorescence corresponds to DAPI, red to laminin, and green to NPS-P88. Scale bars: 50 μm. The PCC r value for the merged signal of laminin and NPS-P88 is shown in the upper right corner. Histograms representing PCC for colocalization of laminin and NPS-P88 in T98G cells are presented at 3 (E) and 24 hours (F). The mean PCC for T98G cells without stimulation at 3 hours was r = 0.278, while for T98G cells with IL-1β was r = 0.426. The mean PCC for T98G cells without stimulation at 24 hours was r = 0.119, while for T98G cells with IL-1β was r = 0.071. PCC (summarized signal) values >0.5 indicate a high probability that pixels of both channels overlay. The data are expressed with normalized mean values±SEM in arbitrary units (a.u). (n = 2 for panels E and F). Student t-test analysis.

Surprisingly, NPS-P88 presented positive labeling in T98G cells at 3 hours in IL-1β stimulated and unstimulated cells, although to a lesser extent in stimulated cells (Fig 4A and 4B). However, no correlation was found between laminin and NPS-P88 in unstimulated cells (r = 0.278±0.067) nor cells under inflammation for 3 hours (Fig 4E; r = 0.426±0.055).

The labeling of NPS-P88 was also examined at 24 hours with or without IL-1β in T98G cells (Fig 4C and 4D). No correlation was found between laminin and NPS-P88 labeling after 24 hours either when the cells were unstimulated (r = 0.119±0.000), or when stimulated with IL-1β (r = 0.071±0.0319) (Fig 4F).

Based on the results obtained, we decided to evaluate the expression of laminin β1 in hCMEC/D3 and T98G cells and corroborate the findings by immunofluorescence after 3 hours, where significant differences and correlation between NPS-P88 and laminin were found. Additionally, we assessed the expression of IL-1β to confirm cell inflammation.

**Fig 5:**
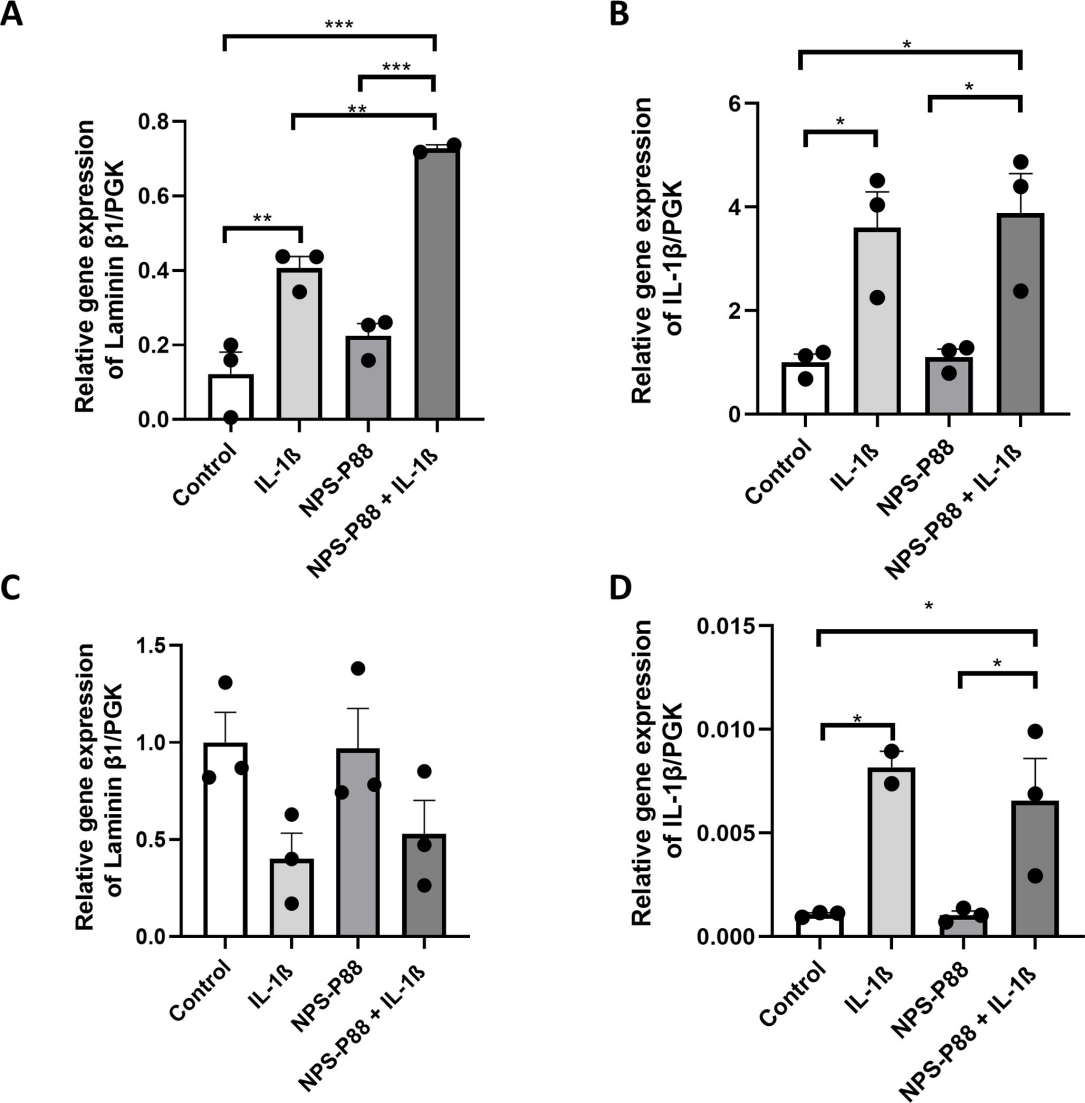
Expression of laminin β1 and IL-1β in hCMEC/D3 and T98G cells by rt-PCR at 3 hours. A) Expression of laminin β1 in hCMEC/D3, B) Expression of IL-1β in hCMEC/D3, C) Expression of laminin β1 in T98G, D) Expression of IL-1β in T98G. * p < 0,05; ** p < 0,01; *** p < 0,001 (n = 2–3 for panels A and D; n = 3 for panels B and C). ANOVA analysis with Tukey’s post hoc test.

In Fig 5A, an increase in laminin β1 expression in hCMEC/D3 cells during inflammatory stimuli for 3 hours (p<0.01; 0.406±0.03) compared with unstimulated cells (0.12±0.05) was evident. Furthermore, a significant increase in laminin β1 expression was observed when cells were incubated with NPS-P88 and stimulated with IL-1β for 3 hours (p<0.001; 0.72±0.009) compared with cells incubated with NPS-P88 alone (0.22 ± 0.03), unstimulated cells (p<0.001; 0.12±0.05), and cells incubated only with IL-1β (p<0.01; 0.406±0.03). These results agree with the findings obtained by immunofluorescence, indicating an increase in laminin expression during inflammatory processes. In Fig 5B, the results of IL-1β expression are shown, confirming an increase in IL-1β in cells under inflammatory conditions for 3 hours (p<0.05; 3.60±0.68) compared with unstimulated cells (1.00±0.16). In addition, an increase in IL-1β expression was confirmed in hCMEC/D3 incubated with NPS-P88 during inflammatory processes (p<0.05; 3.88±0.76) compared with cells incubated only with NPS-P88 (1.102±0.15). No differences were found between unincubated cells and cells incubated with NPS-P88, and there were no differences between cells undergoing inflammatory processes without NPS-P88 and cells undergoing inflammatory processes with NPS-P88.

Furthermore, statistical analyzes indicate no significant difference in laminin β1 expression in T98G cells incubated with or without NPS-P88, stimulated with or without IL-1β for 3 hours (Fig 5C), cells without IL-1β (1,00±0,15), cells with IL-1β (0,4±0,13), cells with NPS-P88 (0,96±0,20), or cells with NPS-P88 and IL-1β (0,52±0,17). In Fig 5D, the results of IL-1β expression are shown, confirming an increase in IL-1β in cells under inflammatory conditions for 3 hours (p<0.05; 0.008±0.0007) compared with unstimulated cells (0.001±0.00007). Additionally, an increase in IL-1β expression was confirmed in T98G cells incubated with NPS-P88 during inflammatory processes (p<0.05;0.006±0.002) compared with cells incubated only with NPS-P88 (0.001±0.00019). No differences were found between unincubated cells and cells incubated with NPS-P88, and there were no differences between cells undergoing inflammatory processes without NPS-P88 and cells undergoing inflammatory processes with NPS-P88.

**Fig 6:**
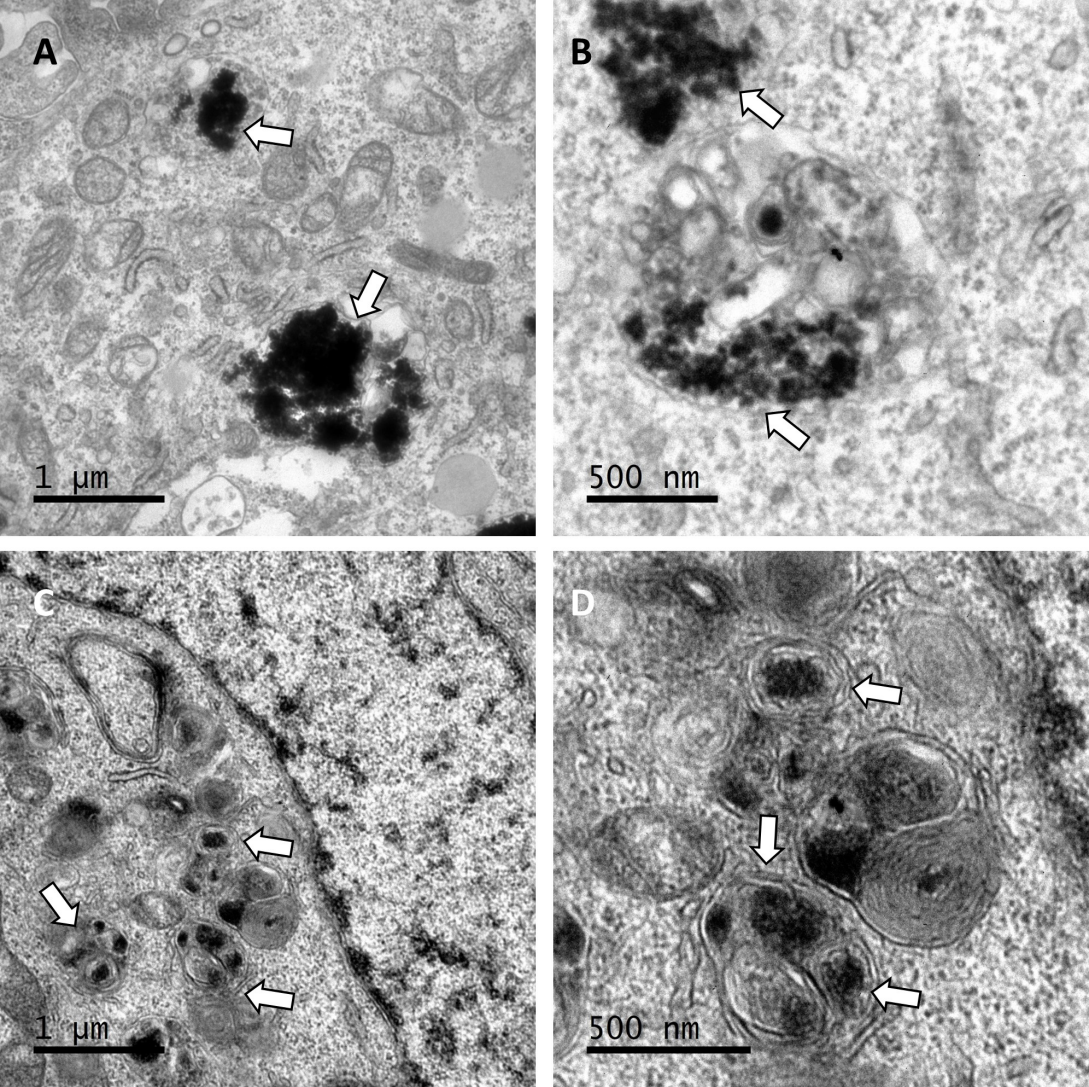
Confirmation of NPS internalization in cells after a three-hour incubation period by TEM. A-B) hCMEC/D3 cells, C-D) T98G cells. White arrows indicate the presence of NPS. Scale bars: 1 μm (A and C); 500 nm (B and D).

In the TEM images obtained from hCMEC/D3 and T98G cells after a 3-hour incubation with NPS, it was observed that in hCMEC/D3 cells, the NPS-P88 were internalized by the cells. They were primarily located in proximity to the cell membrane and also within the cytoplasm, often clustered together (Fig 6A and 6B). Images were also captured from T98G cells, revealing that the NPS-P88 were primarily localized within the cytoplasm, exhibiting signs of encapsulation (Fig 6C and 6D).

### 2.4 *In vivo* assays

The pharmacokinetic results obtained in a female Wistar rat are shown in S5A Fig. The blood iron concentration reached its highest point 30 minutes after the injection of the NPS-P88, compared to the basal concentration. After one hour, the Fe concentration decreased to a control state and remained low at 6 and 24 hours. Therefore, it can be inferred that the increase in Fe at 30 minutes was due to the NPS-P88 and not caused by other variables. Furthermore, an increase in Fe can be observed in the urine one hour after the injection of NPS-P88, compared to the basal state. This is consistent with the decrease in Fe in the blood observed at the same time. Additionally, the Fe concentration returns to its basal state after 6 hours and 24 hours (S5B Fig). According to our results, the half-life of these NPS was 1.5 hours using the previously reported half-life equation [60]. Our results agree with those reported in other USPIO studies in rats [61].

Once the pharmacokinetics of NPS-P88 were known, the presence of nanoprobes in the brain was investigated. For this purpose, the NPS were tagged with Prussian blue in the brain at two time points: 30 minutes and 24 hours, following the injection of NPS-P88. Notably, the cortex of the animal’s brain exhibited detectable traces of NPS-P88 merely 30 minutes after injection (S6A Fig). However, the brain displayed no observable labeling of NPS-P88 after 24 hours from the injection of the NPS-P88 (S6B Fig).

**Fig 7:**
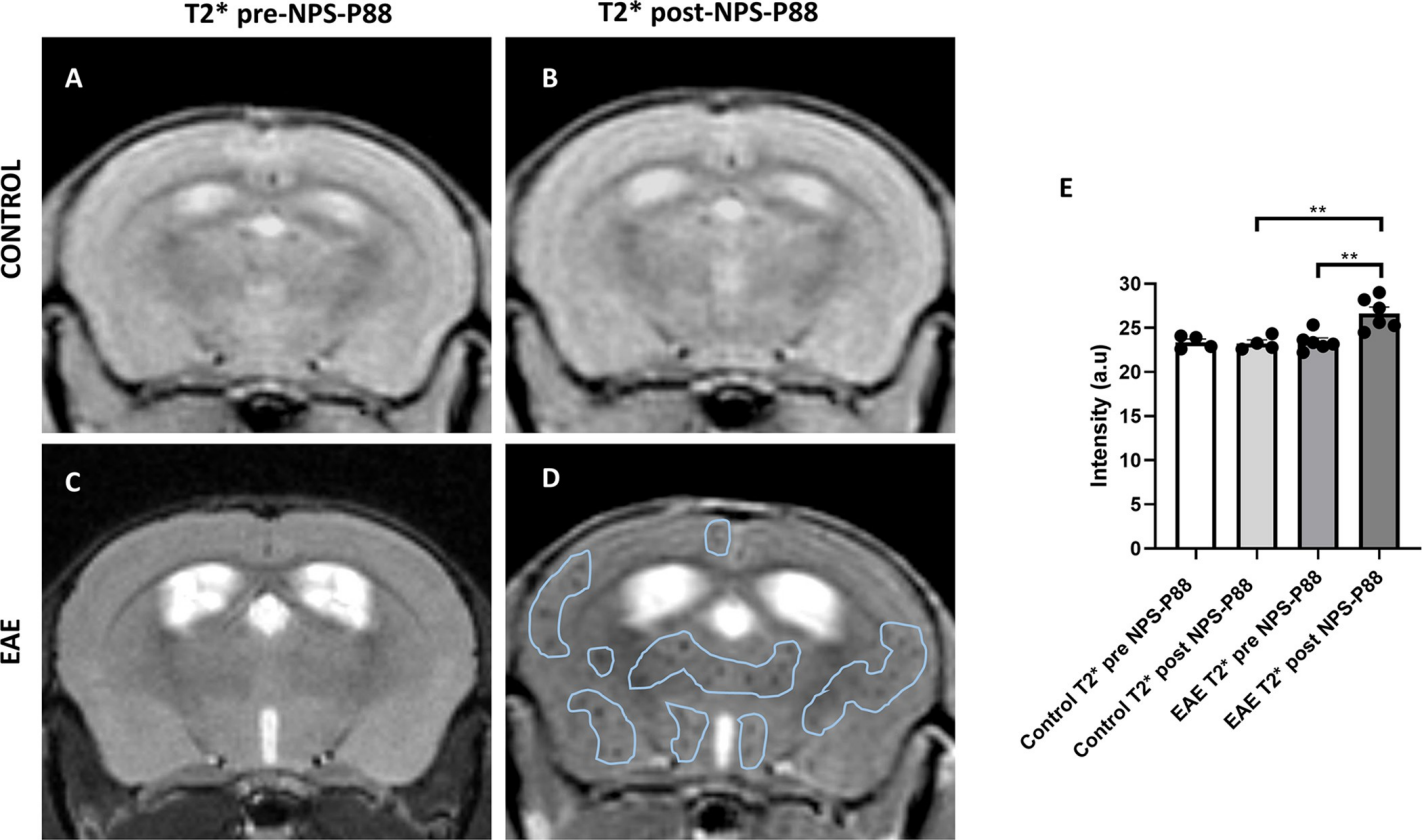
Representative in vivo T2*-weighted MRI images of control mice and mice with EAE before and after NPS-P88 injection. A) MRI image in control animal prior to NPS-P88 injection. B) MRI image in control animal after NPS-P88 injection. C) MRI image in EAE animal prior to NPS-P88 injection. D) MRI image in EAE animal after NPS-P88 injection. E) Quantification of intensity changes in the contrast generated by NPS-P88 in T2* imaging. Comparison intensity between control T2* pre-NPS-P88, control T2* post-NPS-P88, EAE T2* pre-NPS-P88 and EAE T2* post-NPS-P88. ** p < 0.01 (n = 4 for controls, n = 6 for EAE). Analysis was performed using ANOVA analysis with Tukey’s post hoc test.

Finally, MRI experiments were conducted on SJL mice, which were divided into two groups: EAE model induction and control group. T2* images were acquired before and after the injection of NPS-88 to compare the infiltration of NPS-P88 in both control and experimental animals. As depicted in Fig 7B, there was no observation of infiltration of NPS-P88 by MRI 30 minutes after injecting the nanoprobes in control animals, indicating no changes in the before and after images of NPS-P88. Conversely, in the T2* images obtained 30 minutes after injecting NPS-P88 in EAE model, contrast was evident with certain black dots appearing in different areas of the brain, primarily in the cortex (secondary motor cortex, secondary somatosensory cortex, dysgranular insular cortex, granular insular cortex, agranular insular cortex and posterior cortex), thalamic nucleus, cerebral nuclei, and midbrain (Fig 7D). In comparison, no such contrast was observed in the T2* pre-NPSP88 images. Upon comparing the T2* images between the control and experimental groups, it became apparent that the contrast observed was due to the presence of NPS-P88 in the EAE model, whereas the controls did not exhibit such contrast.

When conducting statistical analyses of the intensity generated by the nanoparticle contrast on MRI in T2* scans, no significant difference was observed among the controls before injecting NPS-P88 (23.37±0.369) and 30 minutes after injection of NPS-P88 (23.24±0.388) (Fig 7E). On the other hand, a significant increase in contrast was observed for NPS-P88 injected 30 minutes later in animals with EAE (p = 0.0021; 26.64±0.729) compared to images taken before injecting NPS-P88 in EAE animals (23.44±0.426) (Fig 7E). Furthermore, a significant difference was found when comparing the intensity contrast results in the T2* images taken 30 minutes after NPS-P88 injection between EAE animals (p = 0.0033; 26.64±0.729) and controls (23.24±0.388) (Fig 7E).

Additionally, T1 images were acquired before and after the injection of Gd to assess BBB permeability in both control and experimental animal groups. As illustrated in S7 Fig (see supporting information) on the right, no contrast was observed by MRI 30 minutes after injecting Gd in the control animals when compared to the image obtained before Gd injection. This indicates the stability of the BBB. In contrast, in the T1 images obtained 30 minutes after Gd injection in the EAE model, contrast was evident, with certain areas appearing white, indicating the presence of Gd and a disruption in the BBB. In comparison, no such contrast was observed in the T1 images prior to Gd administration. When comparing the T1 images between the control and experimental groups, it became evident that the Gd-enhanced contrast was due to BBB permeability in the EAE model, whereas the control group did not exhibit this contrast. We conducted statistical analyses of the intensity generated by Gd contrast on MRI in T1 scans and found no significant difference among the controls before injecting Gd (24.36±0.538) and 30 minutes after injection of Gd (27.69±0.307) (S8 Fig). In contrast, a significant increase was noted for Gd injected 30 minutes later in animals with EAE (p = 0.0001; 40.65±1.906) compared to images taken before injecting Gd in EAE animals (24.32±1.047) (S8 Fig). Furthermore, a significant difference was found when comparing the intensity contrast results in the T1 images taken 30 minutes after Gd injection between EAE animals (p = 0.0001; 40.65±1.906) and controls (27.69±0.307) (S8 Fig).

Additionally, we decided to analyze the images obtained in T1 after injecting Gd and T2* following the injection of NPS-P88. What we observed was that in the control group, there was no infiltration of NPS-P88 or Gd, indicating the stability of the BBB. Upon merging these images, no contrast or correlation was found. In contrast, when analyzing the images in EAE animals, we observed infiltration of both NPS-P88 and Gd, indicating BBB disruption (S9 Fig, see supporting information). However, we also noticed that the location of NPS-P88 and Gd was different, suggesting that this difference could be attributed to the specificity of NPS-P88, while Gd indicated infiltration without specificity.

## 3. Discussion

In this work, we present USPIO-APTES functionalized nanoprobes conjugated with peptide (peptide 88) for the specific targeting of laminin in altered BBB endothelial cells exposed to neuroinflammation, which can be monitored through MRI.

Our results indicate that NPS-P88 did not show cytotoxicity at a concentration of 1 mM. Likewise, NPS at different concentrations and silanized with APTES did not generate oxidative stress in endothelial cells after 24 hours of exposure. Hence, we consider that our NPS are safe for use at a 1 mM concentration in biological settings.

The immunofluorescence data indicates increased laminin and NPS-P88 labeling during inflammatory processes induced by IL-1β for 3 hours in endothelial cells. Furthermore, we observed a high laminin/NPS-P88 correlation, consistent with a previously published work [22]. No changes in the fluorescence marking of laminin or NPS-P88 under inflammatory conditions, with respect to controls, were found after 24 hours. However, it was found that the correlation between laminin/NPS-P88 at 24 hours was much lower than the correlation observed at 3 hours post inflammation. This finding indicates that the affinity between laminin and NPS-P88 is specific during inflammatory processes and depends on the expression of laminin, with a high affinity under acute inflammatory processes (3 hours) that decreases over time. In addition, a significant increase in laminin fluorescence marking was also found when cells were incubated with NPS-P88 regardless of whether they were stimulated with IL-1β or not. Although, the peptide was specific for laminin only during inflammatory processes and hence the high correlation reported. The above result may be explained because iron oxide NPS can trigger greater protein production or aggregation [62]. Previous studies reported that depending on the concentration of iron oxide NPS, the expression of extracellular matrix proteins such as collagen IV can be increased [63]. In our case, there was an increase in the expression of laminin, a protein that is mainly responsible for forming networks and generating structural stability, a function that it shares with collagen IV [64]. However, P88 only presented specificity and correlation with laminin expression during inflammatory processes.

Additionally, we decided to test the affinity of P88 with laminin in astrocytes (represented by T98G cells), finding that there was binding of the NPS-P88 in T98G cells. This event may be explained because astrocytes can express tight junction and extracellular matrix proteins, such as laminin, and which can also modify the permeability of the BBB via downregulation of angiogenesis [34, 65].

However, no correlation was found between laminin and P88 under normal conditions or inflammatory processes in T98G cells. This result suggests that P88 has a selective affinity for laminin during acute inflammatory processes in brain endothelial cells rather than in T98G cells. Likewise, the immunofluorescence results were corroborated using the rt-PCR technique and in this way evaluate the expression of laminin during inflammatory processes and also in the presence of NPS. We found that the results were consistent in the two techniques, which confirmed the changes in laminin expression and its correlation with NPS-P88 under inflammatory processes. One possible limitation of our *in vitro* studies regards the use of a static 2D model which may not accurately represent *in vivo* conditions. However, the static *in vitro* cell models may offer important insights into BBB properties and interactions and are useful to test aspects such as cytotoxicity and oxidative stress [66]. Nonetheless, future studies will benefit from more advanced culture models based on microfluidic systems, as they can replicate the dynamic conditions of BBB cells, and also allow to mimic better the architecture and function of the BBB, while offering greater physiological relevance [67].

The brain endothelial cells that form the BBB interact with other cells of the neurovascular unit, such as pericytes and astrocytes. Thus, the interactions of endothelial cells with the extracellular matrix are necessary for the maintenance, development, and regulation of the BBB [68]. Although we performed experiments with two types of cells associated with the BBB, we have a limitation as they were studied separately in 2D *in vitro* models. Therefore, we suggest future studies be conducted in an integrated *in vitro* 3D model, and thus evaluate the interaction of laminin not only with brain endothelial cells but also with astrocytes and pericytes. Such co-culture studies could provide more precise information about the physiological environment of the BBB and provide the possibility of carrying out tests with greater proximity to the functioning of the BBB, especially for studies of metastasis or drug transport [69]. The 3D models are characterized by having a good expression of tight junctions and increased TEER values [70], resembling an *in vivo* system much closer. Likewise, studies based on 3D cultures generate structures organized and supported by an extracellular matrix, which facilitates cell interactions through transmembrane receptors and helps modulate gene expression, including basal lamina proteins such as laminin [71]. In addition, brain organoids can offer further advantages for analyzing diverse aspects of BBB, such as drug penetration, among others [72]. Despite these limitations, we have successfully synthesized iron oxide NPS with superparamagnetic properties, low cytotoxicity and ROS production in hCMEC/D3 cells. Furthermore, we have shown how these NPS can be functionalized with P88, and selectively bind to laminin under inflammatory conditions in a time-dependent manner.

Taking into account the encouraging results we obtained in vitro, the next step was to test our nanoprobes in vivo. First, we conducted pharmacokinetic studies of the NPS to evaluate the duration of exposure. The nanoprobes were administered through the tail vein, and blood and urine samples were collected from Wistar rats at various time points. The findings indicated an initial increase in blood iron concentration at 30 minutes, followed by a gradual decrease to baseline levels. In contrast, urinary iron concentration exhibited an increase after 1 hour, implying NPS excretion. Furthermore, there was a heightened presence of NPS-P88 in the brain 30 minutes after administration. Based on these discoveries, the decision was made to proceed with in vivo MRI tests employing NPS-P88. Observations from MRI revealed a higher number of nanoprobes visible due to the inflammatory disruption of the BBB in animals with the EAE model, particularly in proximity to blood vessels. However, this phenomenon was not observed in control animals. This specificity could be attributed to NPS-P88’s affinity for laminin in brain endothelial cells under inflammatory conditions. Thus, significant distinction in NPS-P88 labeling was observed both in vitro and in vivo, enabling a differentiation between control and inflammatory conditions.

For this reason, we are using the USPIO as a vehicle for P88 that specifically binds to laminin. Thus, what we are seeing through MRI are most probably the changes in the expression of a protein during inflammatory processes. Therefore, we suggest we can use this method to be able to assess these molecular changes over time.

Depending on the size and functionalization characteristics of the iron oxide NPS, they can circulate in the vascular compartments for several hours after injection, with a percentage being phagocyted by macrophage/monocyte easier than other cells such as brain endothelial cells [73]. This facilitates their accumulation in the blood and produces an increase in the intensity of the signal. Therefore, some types of iron oxide NPS have been used as contrast agents for the detection of leaks in blood vessels, as well as for the quantification of relative blood volume in the brain [74].

The iron oxide NPS captured by macrophage/monocyte infiltration facilitates the detection of macrophage accumulation in inflammatory lesions in the brain [12, 75]. Therefore, iron oxide NPS extravasation across the BBB will mainly depend on disruption of endothelial tight junctions in brain parenchymal blood vessels [76]. In our in vivo experiments with an EAE model, the control group did not show apparent labeling with NPS-P88, on the contrary, the experimental group under conditions of neuroinflammation and apparent alteration of the BBB did, showing labeling with the NPS in the brain parenchyma. This highlights the permeation capacity of our nanoprobes mainly under BBB disruption conditions. However, given the specificity of our nanoprobes, it would be of particular interest to perform future experiments on a 3D model of the BBB to evaluate the degree of infiltration and the precise permeation mechanism of these nanoprobes at different times under inflammatory conditions.

The findings reported in our paper open the possibility of using our NPS-P88 as an MRI biomarker, tagging changes in laminin due to neuroinflammation. The NPS-P88 has a promising potential in clinical settings, as many neurological and neurodegenerative conditions, such as MS, AD, Parkinson´s disease and stroke, among others, share the presence of neuroinflammation as a deleterious co-factor compromising both BBB and CNS function [77]. Furthermore, many therapeutic determinations depend on the inflammatory condition of a patient, even in emergency scenarios, where the prospect of a selective biomarker for neuroinflammation, and therefore BBB function, may be of extreme help to support critical decisions.

## 4. Conclusions

In summary, we designed and characterized a functional and non-cytotoxic USPIO-P88 nanoprobe, with selective laminin binding properties, and with the potential to be used as a biomarker under inflammatory conditions. The binding of the NPS-P88 was found to be successful in two different human cell lines such as hCMEC/D3 and T98G cells. Besides, we discovered that 3 hours of IL-1β treatment in hCMEC/D3 cells increased both the immunofluorescence labeling of laminin and high colocalization between laminin and NPS-P88 under inflammatory conditions. This laminin response and nanoprobe interaction at 3 hours was observed only in brain endothelial cells and not in T98G cells. Although, after 24 hours of treatment with IL-1β no changes were observed in laminin immunofluorescence intensity or in the binding with NPS-P88. These findings suggest that NPS-P88 has a higher affinity for endothelial cells during inflammatory processes, but that this affinity decreases over time as laminin expression decreases. The *in vivo* pharmacokinetic and Prussian blue findings show a rise in NPS-P88 levels in blood and brain 30 minutes after nanoprobe injection, gradually decreasing thereafter. Notably, MRI reveals significant differences between control and EAE animals, highlighting considerable NPS-P88 buildup, mainly in the cortex. This accumulation is linked to neuroinflammation and disruption of the BBB. Overall, these P88 functionalized NPS can be used in biological models and could aid in MRI evaluation of BBB changes over time under neuroinflammatory conditions.

## 5. Materials and methods

### 5.1 Nanoparticles synthesis

Iron oxide NPS were synthesized by a coprecipitation method as described elsewhere [78]. Briefly, to synthesize 1 g of iron oxide NPS, 1.6 g of KOH (potassium hydroxide;1.05033.0500; Emsure, Merck) in 70 mL of milliQ water was degassed under vacuum for 30 minutes while stirring. In a three neck round bottom flask, a solution of 0,27 mL of HCl 12 M with 8.63 mL of milliQ water was prepared and degassed for 30 minutes before the dissolution of the Fe salts. FeCl_3_·6H_2_O (2.33 g, 8,6 mmol; Iron(III) chloride hexahydrate; 1.03943.0250; Emsure, Merck) and FeCl_2_·4H_2_O (0.86 g, 4.3 mmol; Iron(II) chloride tetrahydrate; 1.03861.0250 Emsure, Merck) were added to the acidified solution and stirred for 15 minutes. The KOH solution was heated to 40°C and then the Fe solution was added dropwise. Temperature was increased to 80°C for 1 hour under constant stirring. To remove excess reagents, the black color precipitate was decanted (via magnetic separation) and thoroughly transferred to a beaker. The precipitate was washed (with a solution of 1% NaCl in milliQ water to accelerate the decantation), dispersed in an ultrasonic bath (VWR 50T Ultrasonic cleaner) for 10 min and decanted (via magnetic separation). This cycle was repeated three times and the washing continued with milli Q water until no residues were found in the supernatant.

### 5.2 Silanization process

The resulting NPS were silanized to improve their biocompatibility and to introduce amine groups at the surface to facilitate subsequent functionalization steps. The procedure for silanizing the iron oxide NPS was carried out as follows, 100 mg of dry NPS were dispersed in 100 mL of milliQ water during 20 minutes in an ultrasonic bath. Next, 400 μL of NH_3_ (30%) were added to adjust a basic pH [79]. The dispersion was heated up to 60°C and 50 μL of the organosilane (3-aminopropyl)triethoxysilane (APTES) 98% were added. Heating was stopped after 1 hour and the mixture was left to react overnight. Silanized NPS were decanted magnetically, washed with milli Q water and dispersed in an ultrasonic bath. This cycle was repeated at least thrice. To dry bare and silanized NPS, a rotavapor system at 35°C was used [78].

### 5.3 Functionalization process

To obtain nanoprobes with a functional ligand capable of active targeting, the C-terminals (-COOH) of the peptide (P88) biotin-TPMMPETSQRFK were conjugated to the free amine groups (-NH_2_) of silanized iron oxide NPS. To functionalize 10 mg of silanized NPS with 225 mmoles of P881 with a molar ratio Fe:peptide of 400:1, we used a carbodiimide method. 1-ethyl-3-(3-dimethylaminopropyl)-carbodiimide (EDC) and N-Hydroxysuccinimide (NHS) were dispersed in a phosphate-buffer saline solution (PBS) with a pH of 5.7 to activate the peptide C-terminal to conjugate with the amino groups at the NPS surface. However, P88 has an inner amino group (it contains lysine) that could react with the C-terminal of another peptide molecule. To avoid this secondary reaction, EDC and NHS were added in excess (10X and 20X the peptide concentration, respectively). After the peptide was added to this mixture, it was readily added to a suspension of silanized NPS also in PBS at a pH of 5.7 and left to react overnight under magnetic stirring at 200 rpm and room temperature. The obtained NPS-P88 nanobioconjugates were decanted magnetically (via a neodymium permanent magnet), washed with milli Q water and suspended in an ultrasonic bath. The cycle was repeated at least thrice.

### 5.4 Characterization techniques

The HD of the iron oxide NPS in aqueous suspension was determined by DLS using a Zetasizer Nano-ZS (Malvern Instruments, Malvern, UK). NPS were dispersed in milliQ water for 30 minutes using an ultrasonic bath. To determine the diameter and morphology of individual NPS in a dry state, HRTEM imaging was carried out in a JEM—ARM 200F Cold FEG TEM/STEM (Jeol, Massachusetts, USA) operating at 200 kV and equipped with a spherical aberration (Cs) probe and image correctors (point resolution 0.12 nm). For the HRTEM sample preparation, a drop of the suspension was deposited onto an amorphous carbon coated Cu-grid and left until evaporation of the liquid was complete. Successful functionalization of the NPS with APTES was verified using attenuated total reflectance Fourier Transformed Infrared Spectroscopy (ATR-FTIR) aided by a IRTracer-100 spectrophotometer (Shimadzu, Japan). Spectra were collected in the range of 4000–500 cm^−1^ with a spectral resolution of 4 cm^−1^ at room temperature and a total of 80 scans per measurement. To prepare the samples, KBr was dried at 150°C for 5 hours and mixed with dry NPS at a concentration of 1% w/w of NPS. Previous to each measurement, an N_2_-purge was performed to avoid interference with CO_2_ and H_2_O traces. Raman measurements were performed to identify the presence of the different iron oxide crystalline phases using a Raman Spectrometer (Horiba, Xplora) with a 638 nm excitation laser. Because magnetite (Fe_3_O_4_) and maghemite (alpha-Fe_2_O_3_) phases are susceptible to thermal decomposition to hematite, the measurements were performed at 1% of the laser radiation power (150 mW according to the manufacturer). Spectra were collected in the range of 100–1000 cm^−1^ with an acquisition time of 90 seconds. The superparamagnetic behavior of iron oxide NPS was determined at room temperature with the aid of a VSM Lake Shore 7400 at room temperature. The isothermal hysteresis loops were acquired sweeping the magnetic field from − 12 kOe to 12 kOe at room temperature.

### 5.5 USPIO MRI contrast agent

A series of MRI measurements were performed on a 24-well phantom, where 12 wells contained five iron concentrations (in duplicate) dispersed in agarose 0.5%, and two agarose controls. Imaging was performed utilizing a 3-tesla Signa Pioneer MRI scanner, manufactured by GE HealthCare, with a 21-channel brain coil. T1 sequences were acquired with a fixed repetition time of 2000 ms, a fixed echo time of 7 ms, and inversion times of 700, 500, 400, 150, 100, 80, and 50 ms. A matrix size of 224 x 224 was employed, with a slice thickness of 3 mm and a slice gap of 3.3 mm. T2 sequences were acquired with a fixed repetition time of 2000 ms and echo times of 10, 20, 30, 40, 50, 60, 90, 110, 130, 150, and 170 ms. A matrix size of 256 x 212 was employed, with a slice thickness of 3 mm and a slice gap of 3.3 mm. The total intensity of each well was measured using the ImageJ program by selecting a circular ROI of 109 mm^2^ area for each image.

### 5.6 Nanoparticle-peptide conjugation and laminin affinity

We used the dot-blot technique to verify: (a) the conjugation between the iron oxide NPS and the P88, and (b) the affinity of the P88 and its specific target, the protein laminin. As positive control, an antibody specific for the laminin β1 subunit (NB120-6571B, Novus Biologicals, LLC, USA) was used. As negative controls, we used (*i*) a PBS solution, (*ii*) 100 μL of non-conjugated iron oxide NPS at an [Fe] of 4 mM, and (*iii*) a scramble peptide (P37) with no affinity against laminin. Controls and samples were performed in triplicate. The dot-blot experiment was performed as follows: a nitrocellulose membrane was impregnated with recombinant laminin β1 protein antigen (2 μg/mL, NBP2-42385PEP, Novus Biologicals, LLC, USA) in PBS/Bovine Serum Albumin (BSA) (A2153, Sigma) 0.1%, overnight at 4°C. Three washes were performed for five minutes each with a wash buffer (1X, Cat. #WA126; R&D Systems, Minneapolis, MN, USA). Finally, the membrane was blocked with a 5% PBS/BSA solution for one hour at room temperature. The membrane was placed on the Mini-PROTEAN II multiscreen apparatus (Bio-Rad, USA), and 100 μL of the solutions were added, corresponding to each experimental group in triplicate, for 2 hours at 4°C under agitation. Afterwards, three washes were performed with washing buffer, and streptavidin HRP (Cat. # 890803, R&D Systems, Minneapolis, MN, USA) was added at a concentration of 1:200 in PBS 1x/BSA 3% for 1 hour, while stirring at room temperature. Next, the membrane was washed three times with the washing buffer, and a chemiluminescent substrate was added (Westernsure Premium Chemiluminescent Substrate, LICOR), mixing solutions A and B in a 1:1 ratio. The capture of the dot blot was performed using the C-DiGit® Blot Scanner (LICOR), and the image was acquired with the Image Studio Digits version 5.2 software, selecting the high sensitivity parameter for image capture.

### 5.7 hCMEC/D3 culture

We used the immortalized Human Cerebral Microvascular Endothelial Cell Line (hCMEC/D3; Cat. #CLU512, CELLutions Biosystems). These cells are prepared from cerebral microvessel endothelial cells (CECs) by transduction with lentiviral vectors carrying the SV40 T antigen and human telomerase reverse transcriptase. In addition, the hCMEC/D3 depict a spindle-shaped, elongated morphology similar to primary cultures of brain endothelial cells, express several brain endothelial markers, express adherence junction and tight junction proteins, as well as functional ABC transporters typical to brain epithelium. For these reasons, the hCMEC/D3 cells have been frequently used as a BBB cell line model [80]. hCMEC/D3 were grown as a confluent monolayer in T25, T75 flasks or cell culture plates (Fisher, UK), pre-coated with 5% collagen diluted in sterile water (Life Technologies) at 37°C and 5% CO_2_ for one hour. The cells were cultured in endothelial basal medium-2 (EBM-2) (Cat. #00190860, Lonza), containing 5% fetal bovine serum (FBS; Cat. #S181B-500, Biowest, USA), 10 mM HEPES (Cat. #BE17-737E, Lonza), 1 ng/mL human fibroblast growth factor (hFGF; Cat. #CC-4068, Sigma-Aldrich), 1.4 μM hydrocortisone (Cat. #4093, Tocris), 5 μg/mL ascorbic acid (Cat. #4055, Tocris), 1/100 Chemically Defined Lipid Concentrate (Cat. #11905031, Gibco™), and 1% antibiotics (penicillin-streptomycin, Cat. #09-757F, Lonza). The culture medium was changed every three to four days. A passage was performed when cells reached between 70–90% confluence. The cells were used for experiments approximately between passages 30–35. Cell counting and cell viability determination were performed with the Countess II FL Automated Cell Counter (Invitrogen, Life Technologies Corporation, WA, USA).

### 5.8 T98G (astrocyte) culture

We cultured T98G (NCI-PBCF-CRL1690; ATCC CRL-1690) human glioblastoma multiforme cells, which were kindly given by the computational biochemistry laboratory of the Faculty of Nutrition and Biochemistry from the Javeriana University (Bogotá campus, Colombia). T98G cells are fibroblast-like cells isolated from the brain of a glioblastoma multiforme white, 61-year-old, male patient, and have been used as a human model for astrocytes, as they express proteins such as GFAP and vimentin [81]. However, T98G cells do have some differences with regular astrocytes, such as a distinct expression of laminin isoforms, which is a limitation of studies using this cell line [82, 83]. The T98G cells were cultured in T25 flasks or plates at 37°C and 5% CO_2_, in Dulbecco’s Modified Eagle Medium (DMEM; Cat. #12614Q, Lonza), supplemented with 10% FBS (Cat. #S181B-500, Biowest, USA) and 1% antibiotics (penicillin-streptomycin, Cat. # 09-757F, Lonza). The medium was changed every two or three days. A passage was performed when cells reached between 70–90% confluence. The cells were used for experiments approximately between passages 18–30. Cell counting and cell viability determination were performed with the Countess II FL Automated Cell Counter (Invitrogen, Life Technologies Corporation, WA, USA).

### 5.9 Cytotoxicity assay (lactate dehydrogenase assay)

The CyQUANT lactate dehydrogenase (LDH) Cytotoxicity Assay Kit (Cat. #C20301, Invitrogen) was used to determine the cytotoxicity of the NPS. The hCMEC/D3 cells were incubated in a 96-well culture plate (2 x 10^4^ cells/well, in triplicate), in the following experimental groups: silanized NPS (1, 2 and 4 mM), NPS conjugated with P88 (1, 2 and 4 mM), and P88 (15 μM), in addition, the following controls were used: cells without treatment, medium alone and the kit controls. Once the cells reached 70%-90% confluency, the unconjugated or conjugated NPS with P88, and P88 in their corresponding concentrations were added, and were left to incubate for 24 hours at 37°C and in 5% of CO_2_. Forty-five minutes before the end of the NPS incubation time, 10 μL of sterilized water and 10 μL of 10X lysis buffer from the kit were added. Once the incubation time was over, 50 μL of medium from each well were transferred to a new 96-well plate. Then, 50 μL of Reaction Mixture, composed of assay buffer stock solution and Substrate Stock Solution from the kit were added to each well, incubated for 30 minutes, and shaked at room temperature in the dark. Finally, 50 μL of stop solution was added to each well and gently mixed. The absorbance reading was performed in an ELISA plate reader (Multiskan FC—SkanIt Software for Multiskan FC, Thermo Scientific) at 450 nm and 620 nm.

### 5.10 Cellular ROS detection assay

The production of reactive oxygen species (ROS) and superoxides in hCMEC/D3 cells (passage 31 with a viability of 92%), was measured using the Cellular ROS/Superoxide Detection Assay Kit (Cat. #ab139476, Abcam), following the manufacturer’s protocol. This kit provides a specific assay for the real-time measurement of global levels of total ROS, and of superoxides, in living cells. Cells were seeded in 96-well plates, 1.5 x 10^4^ cells/well. Briefly, the hCMEC/D3 were incubated with the silanized NPS with APTES at 1, 2, and 4 mM concentrations, using six replicates per group, for 24 hours at 37°C and in 5% of CO_2_. Next, the medium was removed and washed three times with sterile PBS (Cat. #17-516Q, Lonza). Negative control samples were pretreated with a ROS inhibitor (N-acetyl-L-cysteine) 30 minutes prior to stimulation at 37°C and in 5% CO_2_. Wash buffer was added to the other groups. Once the time had elapsed, another 100 μL of ROS/superoxide detection reagent was added to all the wells, and in addition, 10 μL pyocyanin at a concentration of 500 μM was placed in the positive control groups. Finally, all wells were incubated for 1 hour at 37°C in 5% CO_2_. The fluorescence was detected using a fluorescence microplate reader (Cytation3 with Gen5 software, BioTek Instruments) at excitation/emission 488/520 for ROS (standard fluorescein filter setting) and Ex/Em 550/620 nm for superoxides (standard rhodamine filter settings).

### 5.11 Immunofluorescence

The hCMEC/D3 cells (passage 32 with a viability of 87%) and the T98G cells (passage 22 with a viability of 86%) were seeded in 24-well plates, with round cover glass coverslips (#1.5 thickness, 10 mm, Cat. #64–0718, Harvard Apparatus) at a density of 5 x 10^4^ cells/well (in duplicate or triplicate). The round coverslips were previously coated with poly-L-Lysine (Cat. #0413, Sciencell) for T98G cells (2 μg/cm^2^), or with collagen for hCMEC/D3 cells (Cat. #3443-100-01, Cultrex, R&D Systems). Once they reached confluence, these cells were stimulated with or without 20 ng/mL of IL-1β (Cat. #579404, Biolegend) for three or 24 hours, at 37°C in 5% CO_2_.

Once the stimulation was finished, the NPS conjugated with P88 (1 mM) diluted in the culture medium, were added to the wells with the cells, and incubated for three hours at 37°C in 5% CO_2_. After the incubation time, the cells were washed three times with PBS, and fixed in 4% paraformaldehyde for 30 minutes at room temperature, followed by three five-minute washes with PBS. Finally, they were saturated for one hour with 3% goat serum (ab7481, Abcam) in PBS and permeabilized with 0.3% Triton-X 100 (T8787; Sigma Aldrich). The cells were incubated with primary antibodies overnight at 4°C under constant agitation. The following primary antibody and concentration was used for the detection of laminin (1:500; rabbit laminin antibody, Cat. #NB300-144, Novus Biologicals LLC, USA). Cells were then washed three times for five minutes with PBS and incubated with goat pAb to rabbit IgG Alexa fluor 594 (Cat. #ab150080, Abcam), secondary antibody. The P88-biotinylated peptide was detected with Alexa Fluor® 488 streptavidin (2 μg/ml, Cat. #016-540-084, Jackson immunoresearch). Finally, after the antibodies incubation, the cells were washed three times with PBS and mounted with DAPI fluorescent medium (Fluoroshield™ with DAPI, Cat. #F6057, Sigma-Aldrich) in microscope slides (Fisherbrand™ Superfrost™ Plus Stain Slides, Cat. #22-034-979, Fisher Scientific). All assays were performed on cells stimulated under inflammatory conditions (IL-1β) vs. controls.

Immunofluorescence images were obtained with the HC PL FLUOTAR L 40x/0.60 DRY objective, using the Leica DMi8 microscope (Leica Microsystems, Germany) and the Leica Application Suite X (LAS X) version 3.7.5.24914 software. The camera settings used were as follows: Camera (DFC7000T-0056173616), Format Bin1x1 (1920x1440), Digitization (8-bit), Quality Mode (40 MHz), Color Capture Mode (Composite), Live Format Bin1x1 (1920x1440), TXR (EX: 540–580, DC:585, EM:592–668), DAPI (EX:327–383, DC:400, EM: 435–485), and FITC (EX: 460–500. DC: 505, EM: 512–542) channels were used. Three to five random images per field were taken by the same investigator. To analyze the images, the program ImageJ version 1.53q java 1.8.0_172(64-bit) (NIH, USA) was used. For colocalization/correlation analyses, the Pearson correlation coefficient (PCC) was used with the Just Another Colocalization Plugin (JACoP) plugin in ImageJ [84].

### 5.12 RNA isolation and RT-PCR

The hCMEC/D3 cells and the T98G cells were seeded in 12-well plates at a density of 3,5 x 10^4^ cells per well. Once they reached confluence, these cells were stimulated with or without 20 ng/mL of IL-1β (Cat. #579404, Biolegend) for three hours at 37°C in 5% CO_2_. After the stimulation period, the NPS conjugated with P88 (1 mM) diluted in the culture medium, were added to the cell wells and incubated for three hours at 37°C in 5% CO_2_. Total RNA was isolated using TRIzol Reagent (Cat. #15596026, Invitrogen) and reverse-transcribed into first strand cDNA using high-capacity cDNA Reverse Transcription kit with RNase Inhibitor (Ref. 4374966, Thermo Fisher), according to the manufacturer’s instructions. For PCR amplifications, the following reagents were added: 2,5 mL SsoAdvanced Universal SYBR Green supermix (Cat. #1725270, BioRad), 0.5 μL primer R, 0,5 μL primer F, and 0,5 μL RNAse free water (Cat. #351-029-101, Quality biological). PCR conditions were as follows: the thermal profile was 1 cycle of 95°C for 2 minutes for initial denaturation and polymerase activation, and 40 cycles of 95°C for 5 seconds (denaturation), with annealing temperature of 58°C for 30 seconds, and elongation at 72°C for 30 seconds. Melting curve analysis was subsequently performed at 65°C for 5 seconds and at 95°C for 5 seconds to verify assay specificity. RT-qPCR reactions were performed using the CFX Opus 96 thermal cycler (BioRad). The primers used for laminin β1 and IL-1β were synthesized by Macrogen. PGK1 (phosphoglycerate kinase 1) was employed as the housekeeping gene. The sequences of the primers are as follows: PGK1 Forward: CGGGTCGTTATGAGAGTCG; PGK1 Reverse: AATTTGATGCTTGGGACAGC; IL-1β Forward: TGGCTTATTACAGTGGCAATGA; IL-1β Reverse: CGGAGATTCGTAGCTGGATG; Laminin β1 Forward: AGCTGCAAGGGTGACTCC; Laminin β1 Reverse: AGGACGCTGTCGATCCAG.

### 5.13 *In vitro* TEM

The hCMEC/D3 cells and the T98G cells were seeded in 6-well plates at a density of 3,5 x 10^4^ cells per well. Once they reached confluence, these cells were stimulated with or without 20 ng/mL of IL-1β (Cat. #579404, Biolegend) for three hours at 37°C in 5% CO_2_. After the stimulation period, the NPS conjugated with P88 (1 mM) diluted in the culture medium, were added to the cell wells and incubated for three hours at 37°C in 5% CO_2_. Following the incubation time, the cells were washed three times with PBS and were trypsinized and centrifuged at 1200 rpm for 10 minutes. Then, the pellet was diluted in glutaraldehyde 2.5%. Once the cells were fixed, they were centrifuged at 13,000 rpm for 3 minutes. Subsequently, a post-fixation process was carried out using 1% osmium tetroxide in water for two hours at 4°C, followed by a pre-imbibition step with 3% uranyl acetate for 1 hour at room temperature. The dehydration process involved a series of ethanol gradients at different concentrations: 50%, 70%, 90%, 100%, 100%, each lasting 10 minutes. This was followed by acetone-ethanol (1:1) for 15 minutes and then acetone for another 15 minutes. For the SPURRs epoxy resin, the following procedure was followed: Mix Resin Spurr with acetone (2:1) for 1 hour, Mix Resin Spurr with acetone (1:1) for 1 hour, Pure Resin Spurr for 2 hours, and polymerize for 12 hours at 72°C. The samples were sectioned using a Leica EM UC7 ultramicrotome, creating slices with a thickness of 130 nm. These slices were contrasted with 6% uranyl acetate and lead citrate. Finally, the samples were observed using a JEOL 1400 plus TEM, and images were captured using a Gatan Orius CCD camera.

### 5.14 Pharmacokinetics of USPIO

All the experiments comply with the requirements of the Ethics Committee of the Universidad de Los Andes code C.FUA_21–003, (Ley 84/89 y Res 8430/93). For this experiment, a female Wistar rat weighing 250 g was used. Blood and urine samples were obtained 30 minutes prior to the administration of NPS-P88 in order to determine the baseline iron concentration. Subsequently, NPS-P88 were injected into the animal’s tail vein at a concentration of 1 mg Fe/Kg based on previous studies published in murine models [85, 86]. Blood and urine samples were collected 30 minutes, 1 hour, 6 hours, and 24 hours after the injection. Blood collection was performed via the saphenous vein, while urine samples were collected using a platform with perforations to optimize the collection process. Subsequently, the samples were digested by adding 65% nitric acid. Iron quantification was done by atomic absorption with a graphite furnace using a high-resolution continuous source equipment HR-CS-AAS, model CONTRAA800-D (Analytik Jena, Jena, Germany). The working line used was a secondary type (302.0639 nm) with less sensitivity than the primary one, in view of the typical iron concentrations found in the tissues.

### 5.15 Labeling of nanoparticles by Prussian blue

For this determination we used two female Wistar rats, each weighing between 230–250 grams. At time points of either 30 minutes or 24 hours post NPS-P88 injection, an intraperitoneal injection of pentobarbital was performed at a final concentration of 85 mg/kg. Once the animal was completely sedated (in deep sleep), which was confirmed by reflex tests in the lower limbs and eyes, perfusion with PBS and tissue extraction were carried out. The tissues were initially fixed in a 4% PFA solution overnight. Subsequently, they were immersed in a 0.01% NaN_3_ solution in PBS. Thin sections of 30 μm were prepared from both brain and liver tissues, and these sections were stored at -20°C until needed. The tissue underwent a thorough washing process with PBS, involving three five-minute washes. Following this, a solution of 5% potassium ferrocyanide trihydrate (Merck, Cas #14459-95-1) was applied for a duration of 30 minutes. After two additional washes, the tissue was treated with nuclear fast red for a period of 10 minutes. Concluding this step, two PBS washes were made and subsequently covered with a coverslip. Images were observed under a light microscope (Carl Zeiss Primovert Ref: 415510-1101-000).

### 5.16 EAE induction

All animal experiments were approved by the Berlin State Office for Health and Social Affairs (LAGeSo) and conducted in strict adherence to the European guidelines for the care and use of laboratory animals under directive 2010/63/EU of the European Parliament and of the Council of 22 September 2010.

For this experiment, we used Swiss Jim Lambert (SJL) female mice that were 10–15 weeks old (Janvier, SAS, Le Genest Saint Isle, France). The mice were housed under standard conditions with a 12:12 hours light–dark-cycle and *ad libitum* access to food and water. Housing was not randomized. The two experimental groups consisted of female healthy (n = 4) and female EAE (n = 6) mice. The allocation of the animals into either control or experimental group was randomized but not blinded. To induce EAE, the animals were immunized with 250 μg of proteolipid peptide PLP139–151 (The peptide was produced by Peptide synthesis group at Charité), emulsified in 100 μL complete Freund’s adjuvant (Cat # 9225949; Thermo Fisher Scientific, Waltham, MA, USA) and 800 μg Mycobacterium tuberculosis H37Ra (Cat # 231141; Difco, Detroit, MI, USA). Furthermore, 250 ng of pertussis toxin (Cat # LT-0105; List Biological Laboratories, Campbell, CA, USA) resuspended in PBS (Gibco, Grand Island, NY, USA) was injected intraperitoneally on day 0 and day 2 after immunization. EAE induction was not blinded nor randomized. Mice were monitored daily for clinical signs and scored as follows: 0.5—tail paresis or weak righting reflex; 1—tail plegia or tail paresis and weak righting reflex; 1.5—tail plegia and weak righting reflex; 2.0—additional hind limb paresis; 3.0—paraplegia; 4.0—additional forelimb paresis; 5.0—moribund or dead animal. To comply with animal welfare guidelines, all mice with a score greater than 3 would be euthanized and removed from the study. None of the animals met the score, so none were sacrificed.

### 5.17 *In vivo* scans and experimental set-up

*In vivo* scans were performed in controls (without EAE) and in EAE animals with established clinical signs (i.e., animals with at least partial hind limb paresis, score 1.5). For each single animal, imaging post-contrast was compared to the corresponding pre-contrast image. Pre-contrast MRI with a T1-weighted imaging, a T2-weighted sequence to acquire an anatomical image, and a pre-contrast T2*-weighted sequence were obtained. Next, a Gadolinium (Gd) -based contrast agent (GBCA; 0.2 mmol/Kg, Magnevist, Bayer-Schering AG) was administered via the tail vein, and post-contrast T1-weighted images were acquired. Once the scans were completed, 1 mg Fe/Kg USPIO-P88 were intravenously injected into the tail vein, and 30 minutes later, a T2*-weighted sequence was acquired to visualize particle accumulation.

MRI examinations were performed in a preclinical 7 Tesla MRI scanner (BioSpec, Bruker, Ettlingen, Germany) running with ParaVision 6.1 software. All scans were acquired with a 20-mm diameter 1H-RF quadrature volume coil (RAPID Biomedical, Würzburg, Germany). For acquisitions, mice were placed on a custom-built animal holder and anesthetized with 1.5–2.0% isoflurane in 30% O_2_ and 70% N_2_O, administered via an anesthesia mask during continuous respiratory monitoring using a pressure-sensitive pad placed on the thorax (Small Animal Instruments Inc., Stony Brook, NY, United States). Body temperature was kept constant by circulating water through warming pads integrated into the animal holder, and body temperature was monitored using a rectal probe. Therefore, after the last MRI scan, animals were sacrificed with an overdose of ketamine/xylazine.

### 5.18 MRI

Coronal anatomical images were acquired using a T2-weighted 2D-RARE sequence with repetition time (TR) = 3,500 ms, effective echo time (TE) = 33 ms, echo spacing (DTE) = 11 ms, RARE factor = 8, 4 averages, 32 contiguous slices with a slice thickness of 0.5 mm, field of view (FOV) = 18 mm × 18 mm, matrix size MTX = 180 × 180, in-plane resolution 0.1 mm × 0.1 mm × 0.5 mm, bandwidth BW = 34,722 Hz, and total acquisition time TA = 5:08 minutes. GBCA-enhanced images were acquired using a T1-weighted RARE sequence with TR = 800 ms, TE = 6.5 ms, DTE = 6.5 ms, RARE factor = 2, 6 averages, BW = 75,000 Hz, and the same geometry as for the T2w scan resulting in a total acquisition time of TA = 7:12 minutes. To visualize USPIO-P88 accumulation, a T2*-weighted FLASH sequence was used with TR = 400 ms, TE = 2.5 ms, flip angle = 30 degrees, 3 averages, BW = 29,762 Hz, and the same geometry as for the T2w scan with a total acquisition time of TA = 2:24 minutes. The images obtained in T1 and T2* were analyzed before and after the injection of Gd and NPS-P88. Gd contrast is observed in T1, while NPS-P88 contrast is evident in T2*. To conduct the analyses, regions exhibiting contrast due to Gd or NPS-P88 were identified after the injection, and the signal intensity was measured in each. Similarly, the same regions and an area of interest were located in the images obtained before the injection of Gd or NPS-P88 in the corresponding T1 and T2* sequences, enabling subsequent statistical analyses. The analysis of the resonance images was conducted using the ImageJ software.

### 5.19 Statistical analysis

Statistical analysis was performed using GraphPad Prism 8.0 (GraphPad Software Inc., San Diego, CA, USA). Unless otherwise indicated, a *p-*value equal to or less than 0.05 was considered statistically significant for all analyses. To compare between two groups the unpaired Student’s *t*-test was used, and the ANOVA with Bonferroni’s or Tukey’s post hoc tests for three or more group comparisons. The Shapiro-Wilk test was used to analyze the normality of the data. The data are presented as the mean ± standard error of the mean (SEM).

## Supporting information

S1 Fig Characterization of NPS. A) Raman spectra of bare iron oxide NPS using an excitation laser at 632 nm. Magnetite and maghemite peaks are identified by full diamond and open triangle symbols, respectively. Raman intensity is measured in counts per second (cps). B) Dynamic light scattering measurements performed in water for bare iron oxide NPS (open circle, black line) and silanized NPS (open triangle, red line). The mean HD and the PDI are also shown. C) FTIR spectra of KBr pellets of bare iron oxide NPS (top, black line) and silanized iron oxide NPS (bottom, red line). D) Magnetic hysteresis curve of the iron oxide NPS measured at room temperature. Increased magnetic field scan is represented by red triangles pointing up and decreasing magnetic field scan by black triangles pointing down. The inset is a zoom of the figure showing near-zero magnetic coercivity.
(TIF)

S2 Fig Selected area electron diffraction (SAED) pattern of (a) bare and (b) silanized NPS. Red lines indicate the corresponding crystallographic planes of the magnetite phase.
(TIF)

S3 Fig USPIO as MRI contrast agent. A) Magnetic resonance T2-weighted images of USPIO NPS in agarose suspension (0.5% agarose) with different iron concentrations ranging from 0.0 mM to 2.0 mM. Spin ECHO (SE) sequence with TR = 2000 ms and TE = 20 ms. B) Magnetic resonance T1-weighted images of USPIO NPS in agarose suspension (0.5% agarose) with different iron concentrations from 0.0 mM to 2.0 mM. Inversion recovery (IR) sequence with TR = 2000 ms and inversion times (TI) of (a) 50 ms and (b) 700 ms.
(TIF)

S4 Fig A) Effect of changing Time Echo (TE) in *T2*-weighted images of USPIO NPS in agarose suspension (0.5% agarose) with different iron concentrations from 0.0 mM to 2.0 mM. B) Effect of changing Inversion time (TI) in *T1*-weighted images of USPIO NPS in agarose suspension (0.5% agarose) with different iron concentrations from 0.0 mM to 2.0 mM.
(TIF)

S5 Fig Quantification of iron with respect to time after NPS-P88 injection in female Wistar Rat. A) Iron concentration in the blood at different time points after NPS-P88 injection. B) Iron concentration in the urine at different time points after NPS-P88.
(TIF)

S6 Fig Staining with Prussian blue of the brain cortex of a Wistar rat. A) 30 minutes and, B) 24 hours after intravenous injection of NPS-P88. White arrows indicate the presence of NPS in the brain cortex. Scale bars: 100 μm.
(TIF)

S7 Fig Representative in vivo T1-weighted MRI images of control mice and mice with EAE before and after gadolinium (Gd). A) MRI image in control animal prior to Gd injection (left). and MRI image in control animal after Gd injection (right). B) MRI image in EAE animal prior to Gd injection (left) and MRI image in EAE animal after Gd injection (right).
(TIF)

S8 Fig Quantification of intensity changes in the contrast generated by Gd in T1 imaging. Comparison intensity between control T1 pre-Gd, control T1 post-Gd, EAE T1 pre-Gd and EAE T1 post-Gd. ** p < 0.0001 (n = 4 for controls, n = 6 for EAE). Analysis was performed using ANOVA analysis with Tukey’s post hoc test.
(TIF)

S9 Fig Representative in vivo T1, T2* and merge-weighted MRI images of control mice and mice with EAE before and after gadolinium (Gd) and NPS-P88. A) MRI image in control animal post to Gd injection, NPS-P88 injection and Merge. B) MRI image in EAE animal after Gd injection, NPS-P88 and Merge (in red T1 for Gd and green T2* for NPS-P88).
(TIF)

S1 Raw images Original image of the dot-blot assay of laminin-impregnated nitrocellulose membrane. The image presented in Fig 2 is a cropped version of this image, modified to improve the presentation of the results after removing irrelevant lanes and including the labels for each of the columns.
(TIF)

S1 Table Reagents and antibodies. (XLSX)

## Abbreviations

APTES: (3-aminopropyl)triethoxysilane
ATR-FTIR: attenuated total reflectance Fourier Transformed Infrared Spectroscopy
BBB: blood-brain barrier
BMs: basement membranes
BSA: Bovine Serum Albumin
CECs: cerebral microvessel endothelial cells
CNS: central nervous system
DLS: Dynamic Light Scattering
EAE: experimental autoimmune encephalomyelitis
ECM: extracellular matrix
EDC: 1-ethyl-3-(3-dimethylaminopropyl)-carbodiimide
FCC: face-centered cubic
FFT: fast Fourier transform
HD: hydrodynamic diam
HRTEM: high-resolution transmission electron microscopy
IL-1β: interleukin 1β
LDH: lactate dehydrogenase
LRP-1: lipoprotein receptor protein 1
MRI: magnetic resonance imaging
MS: multiple sclerosis
NHS: N-Hydroxysuccinimide
NPS: nanoparticles
NPS: P88 USPIO-P88 nanoprobes
P88: peptide-88
PBS: phosphate-buffer saline solution
PDI: polydispersity index
ROS: reactive oxygen species
SAED: selected area electron diffraction
USPIO: Ultrasmall superparamagnetic particles of iron oxide
VSM: vibrating-sample magnetometer
