# Supplementary figures and images for: Specific nanoprobe design for MRI: Targeting laminin in the blood-brain barrier to follow alteration due to neuroinflammation
Source: PLoS One. 2024 Apr 11;19(4):e0302031. doi: 10.1371/journal.pone.0302031 (PMC11008835; doi:10.1371/journal.pone.0302031)

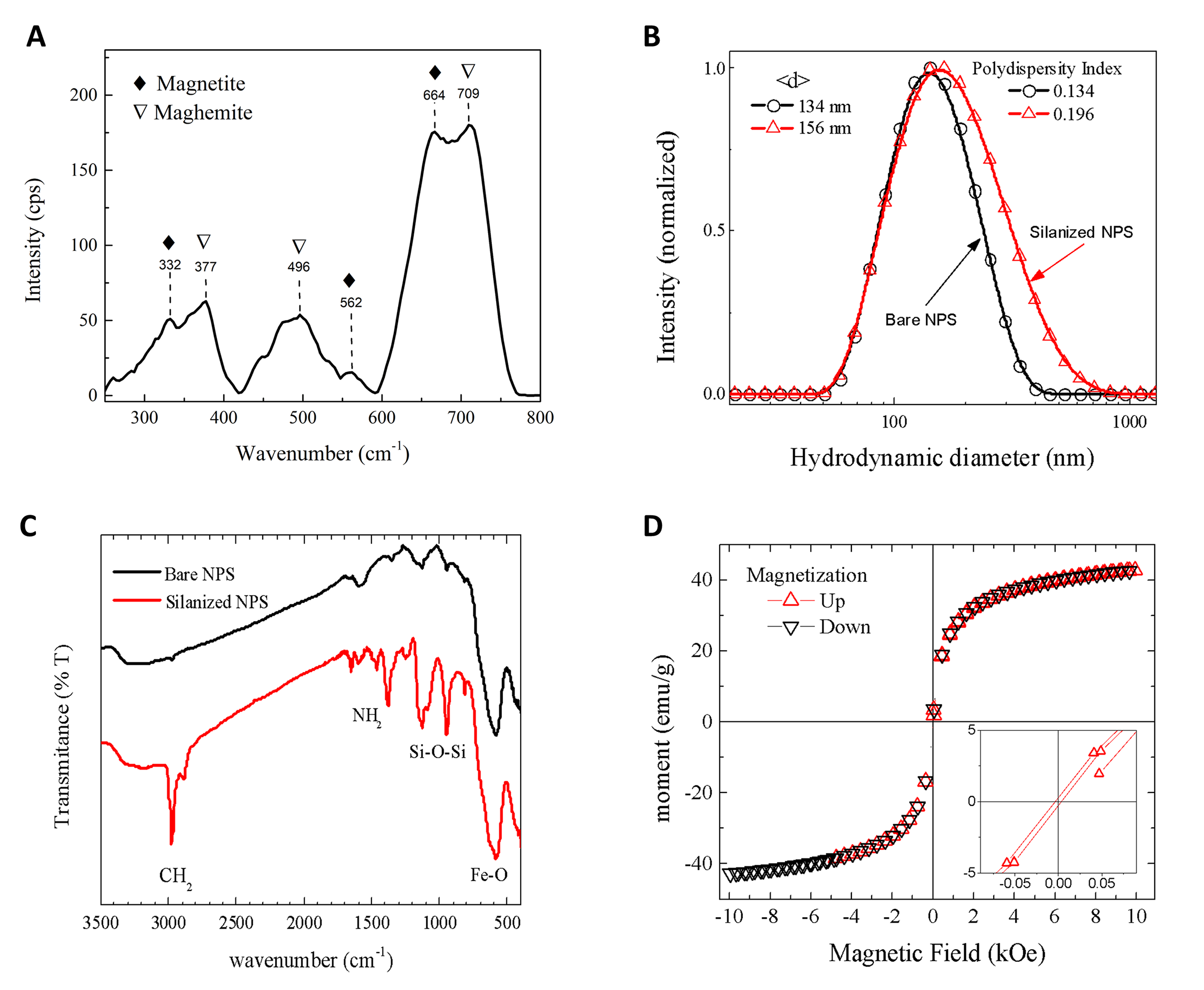

Supplement: S1 Fig — A) Raman spectra of bare iron oxide NPS using an excitation laser at 632 nm. Magnetite and maghemite peaks are identified by full diamond and open triangle symbols, respectively. Raman intensity is measured in counts per second (cps). B) Dynamic light scattering measurements performed in water for bare iron oxide NPS (open circle, black line) and silanized NPS (open triangle, red line). The mean HD and the PDI are also shown. C) FTIR spectra of KBr pellets of bare iron oxide NPS (top, black line) and silanized iron oxide NPS (bottom, red line). D) Magnetic hysteresis curve of the iron oxide NPS measured at room temperature. Increased magnetic field scan is represented by red triangles pointing up and decreasing magnetic field scan by black triangles pointing down. The inset is a zoom of the figure showing near-zero magnetic coercivity. (TIF) [file pone.0302031.s001.tif]

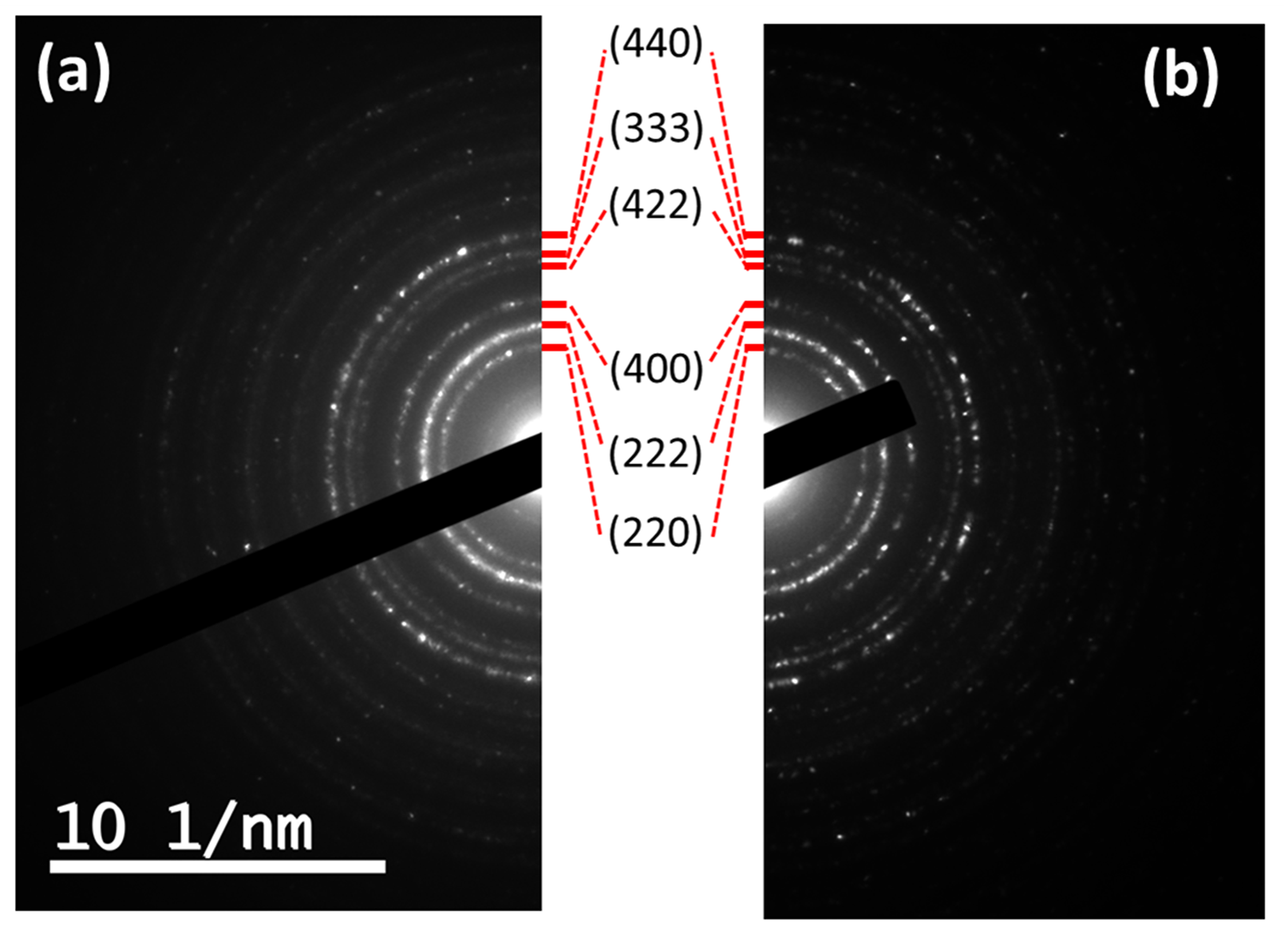

Supplement: S2 Fig — Selected area electron diffraction (SAED) pattern of (a) bare and (b) silanized NPS. Red lines indicate the corresponding crystallographic planes of the magnetite phase. (TIF) [file pone.0302031.s002.tif]

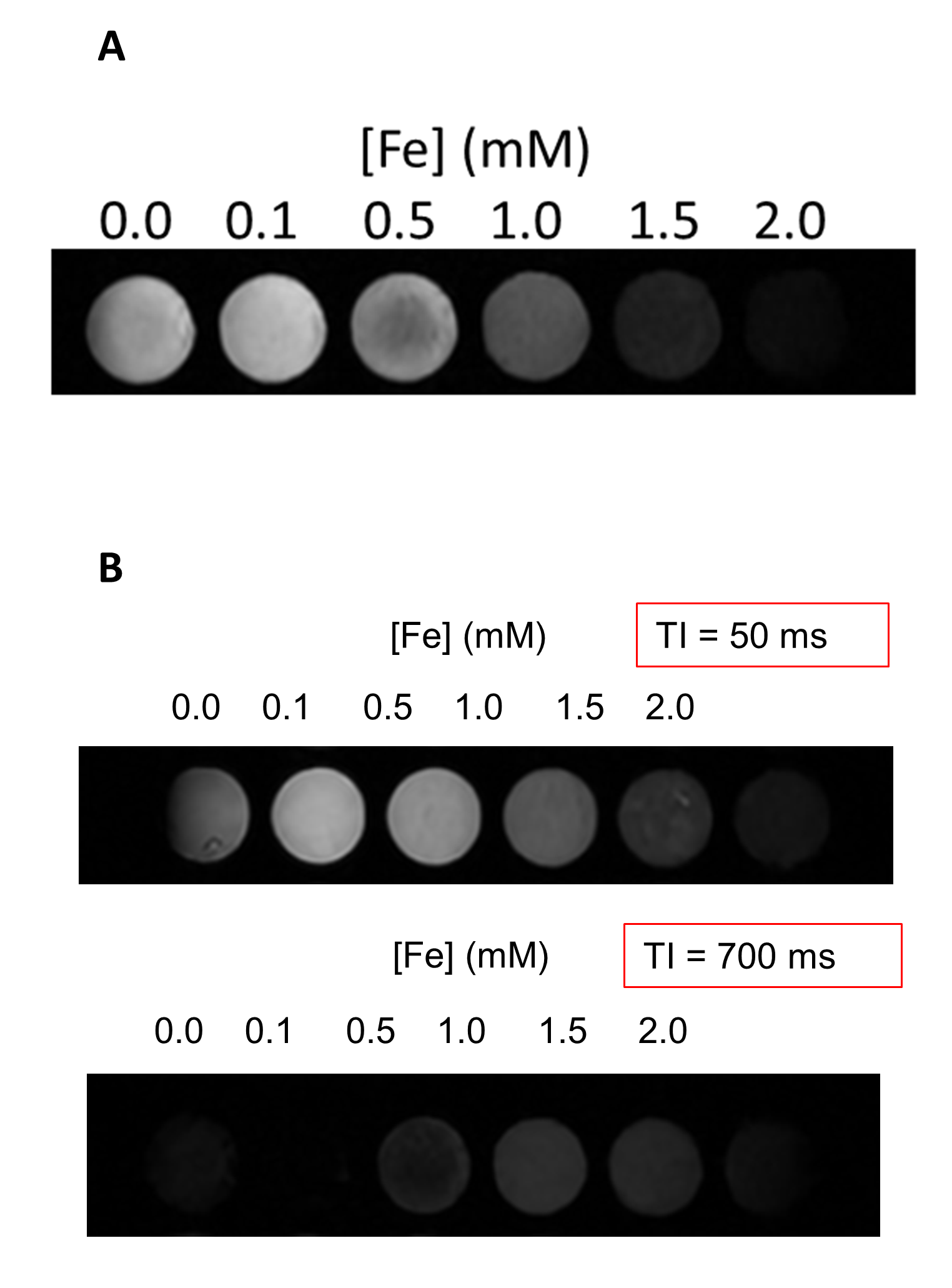

Supplement: S3 Fig — A) Magnetic resonance T2-weighted images of USPIO NPS in agarose suspension (0.5% agarose) with different iron concentrations ranging from 0.0 mM to 2.0 mM. Spin ECHO (SE) sequence with TR = 2000 ms and TE = 20 ms. B) Magnetic resonance T1-weighted images of USPIO NPS in agarose suspension (0.5% agarose) with different iron concentrations from 0.0 mM to 2.0 mM. Inversion recovery (IR) sequence with TR = 2000 ms and inversion times (TI) of (a) 50 ms and (b) 700 ms. (TIF) [file pone.0302031.s003.tif]

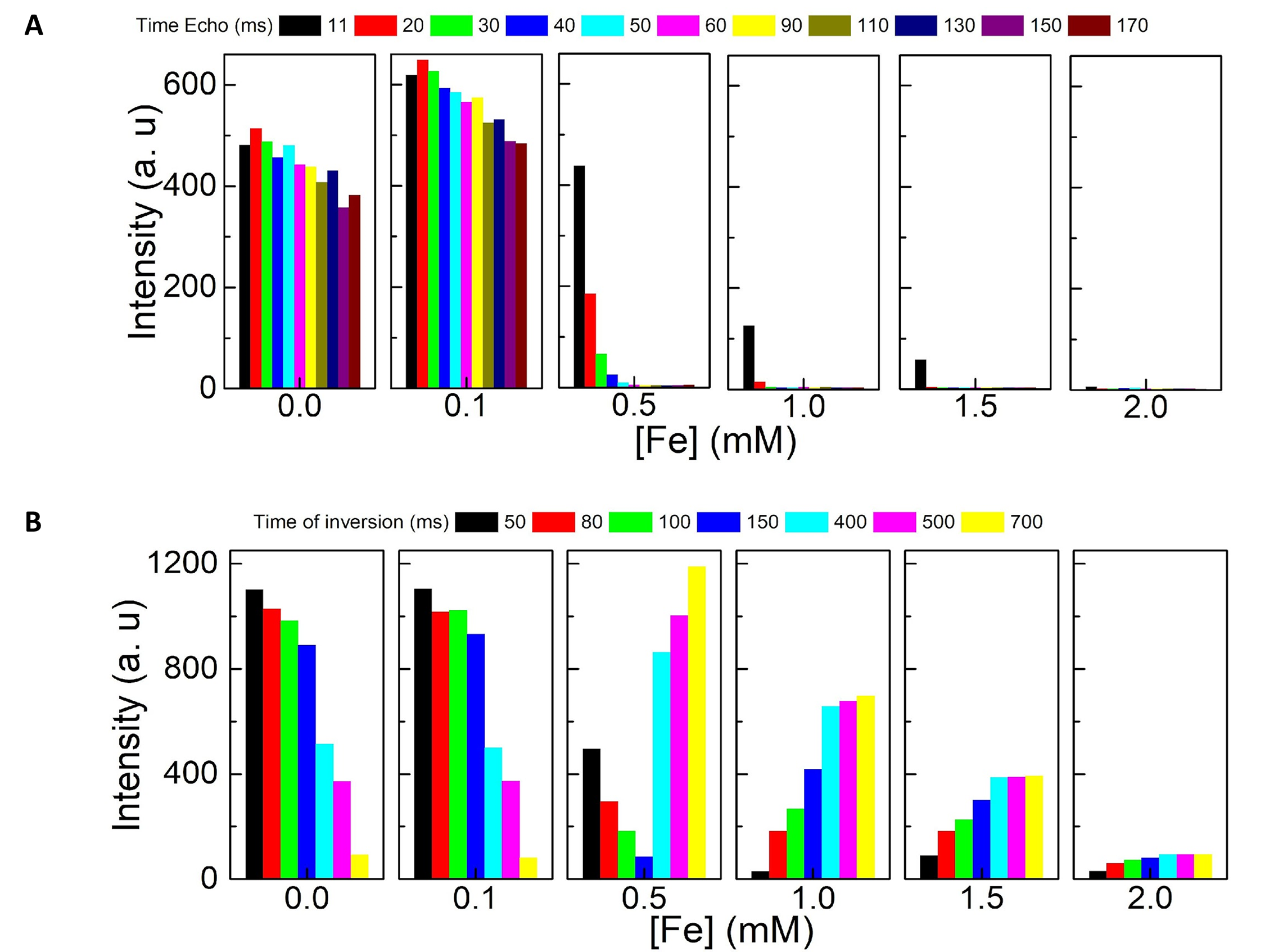

Supplement: S4 Fig — A) Effect of changing Time Echo (TE) in T2-weighted images of USPIO NPS in agarose suspension (0.5% agarose) with different iron concentrations from 0.0 mM to 2.0 mM. B) Effect of changing Inversion time (TI) in T1-weighted images of USPIO NPS in agarose suspension (0.5% agarose) with different iron concentrations from 0.0 mM to 2.0 mM. (TIF) [file pone.0302031.s004.tif]

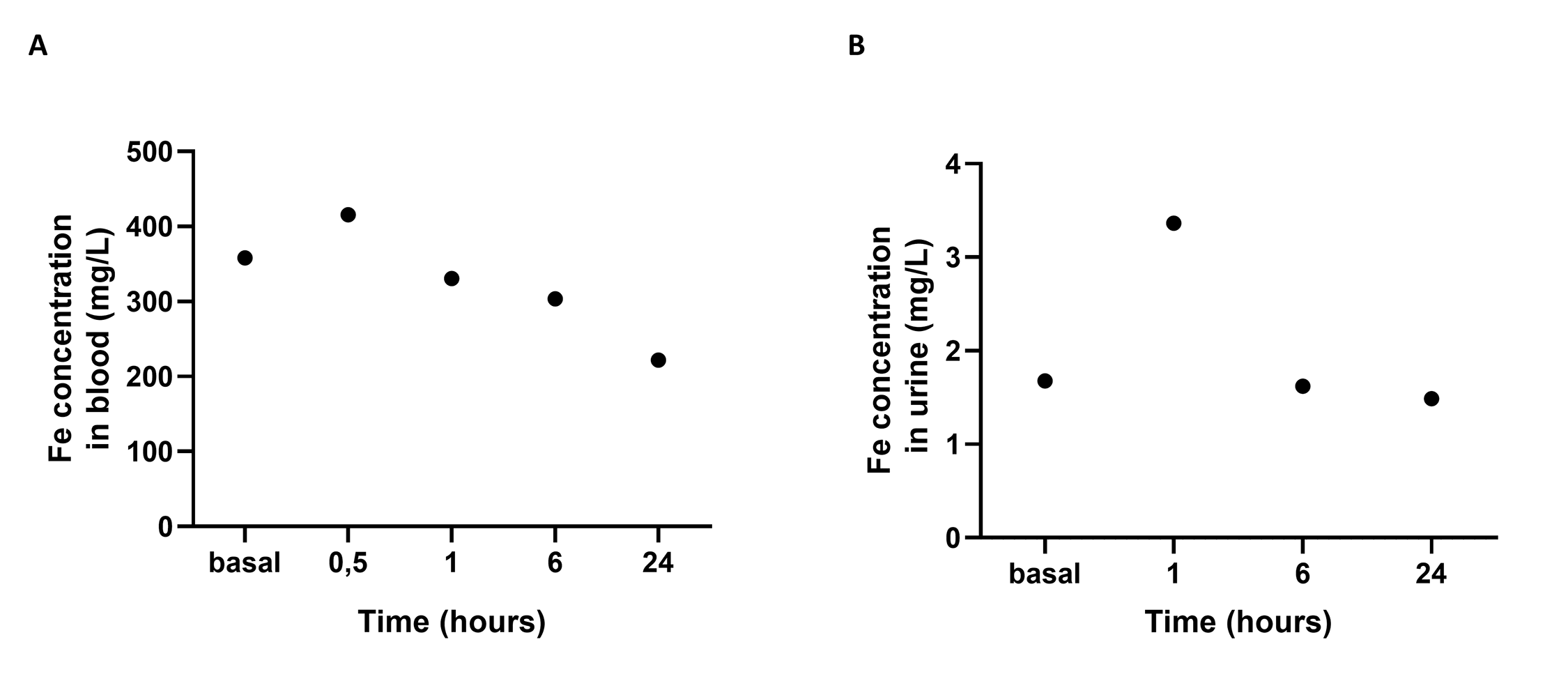

Supplement: S5 Fig — A) Iron concentration in the blood at different time points after NPS-P88 injection. B) Iron concentration in the urine at different time points after NPS-P88. (TIF) [file pone.0302031.s005.tif]

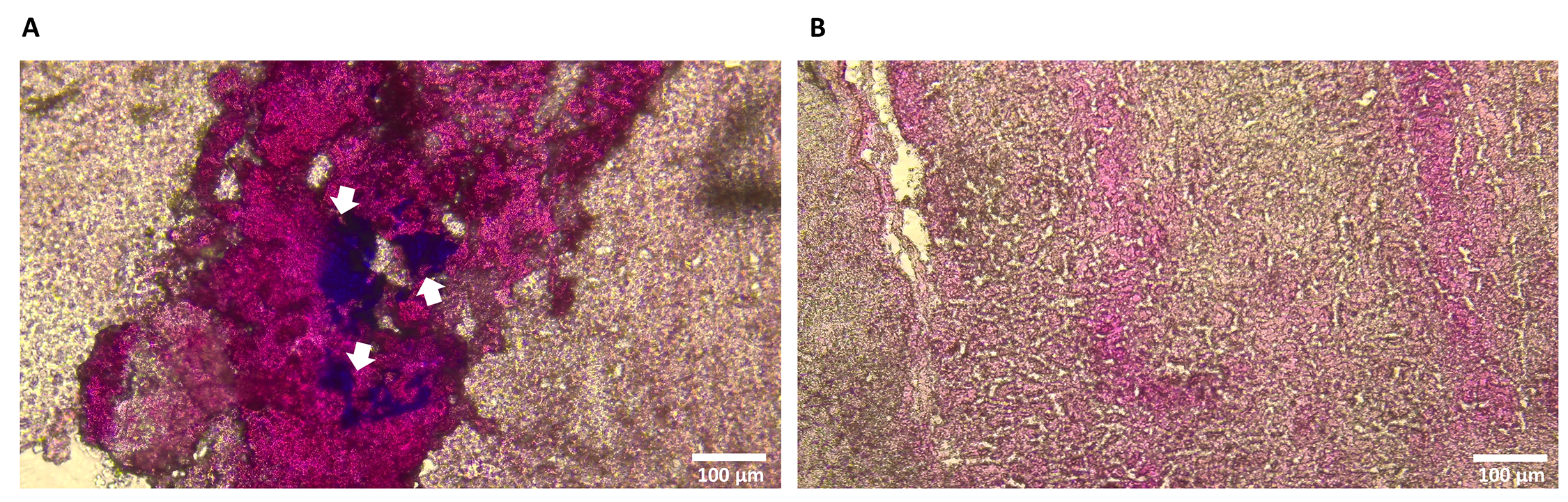

Supplement: S6 Fig — A) 30 minutes and, B) 24 hours after intravenous injection of NPS-P88. White arrows indicate the presence of NPS in the brain cortex. Scale bars: 100 μm. (TIF) [file pone.0302031.s006.tif]

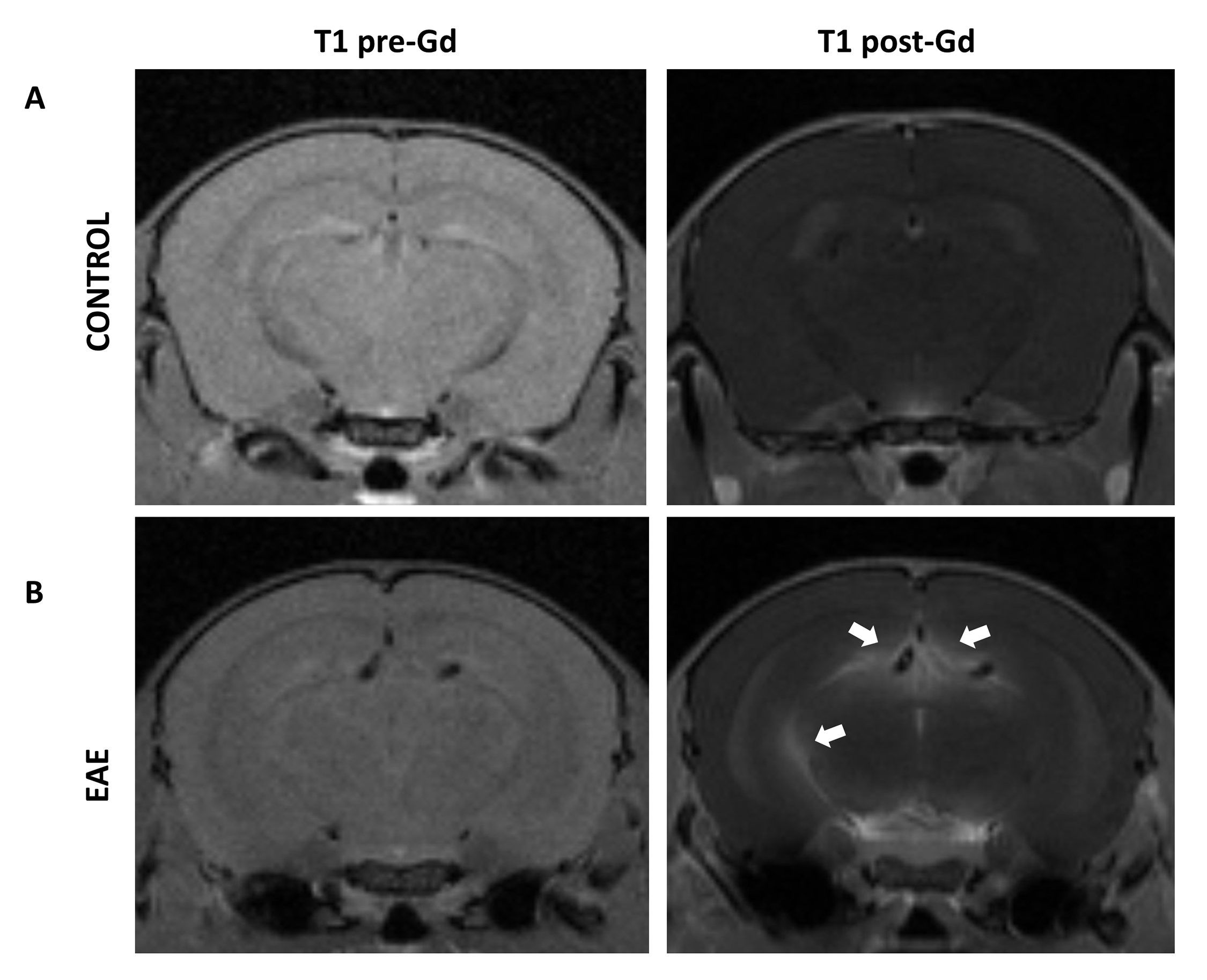

Supplement: S7 Fig — A) MRI image in control animal prior to Gd injection (left). and MRI image in control animal after Gd injection (right). B) MRI image in EAE animal prior to Gd injection (left) and MRI image in EAE animal after Gd injection (right). (TIF) [file pone.0302031.s007.tif]

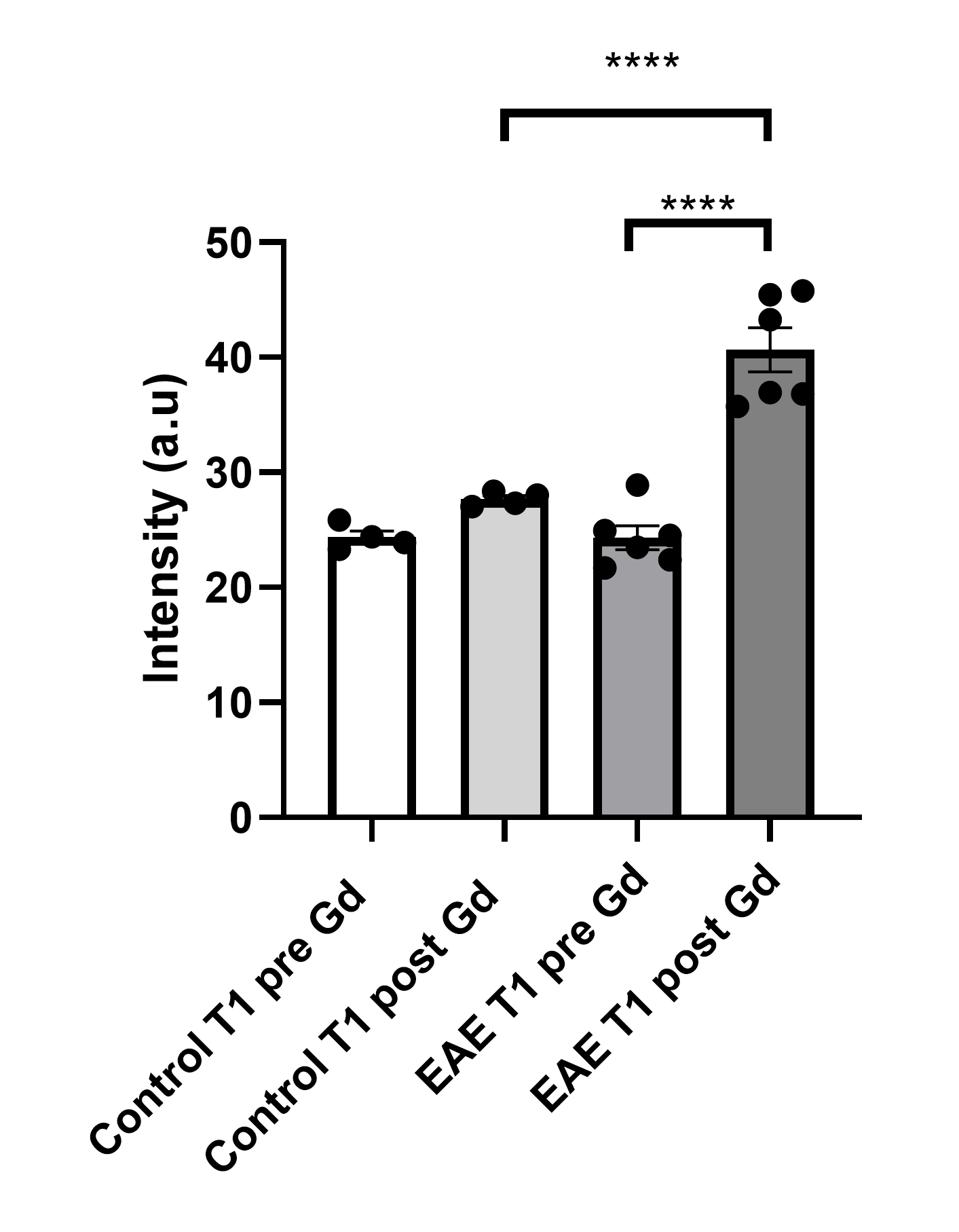

Supplement: S8 Fig — Comparison intensity between control T1 pre-Gd, control T1 post-Gd, EAE T1 pre-Gd and EAE T1 post-Gd. ** p < 0.0001 (n = 4 for controls, n = 6 for EAE). Analysis was performed using ANOVA analysis with Tukey’s post hoc test. (TIF) [file pone.0302031.s008.tif]

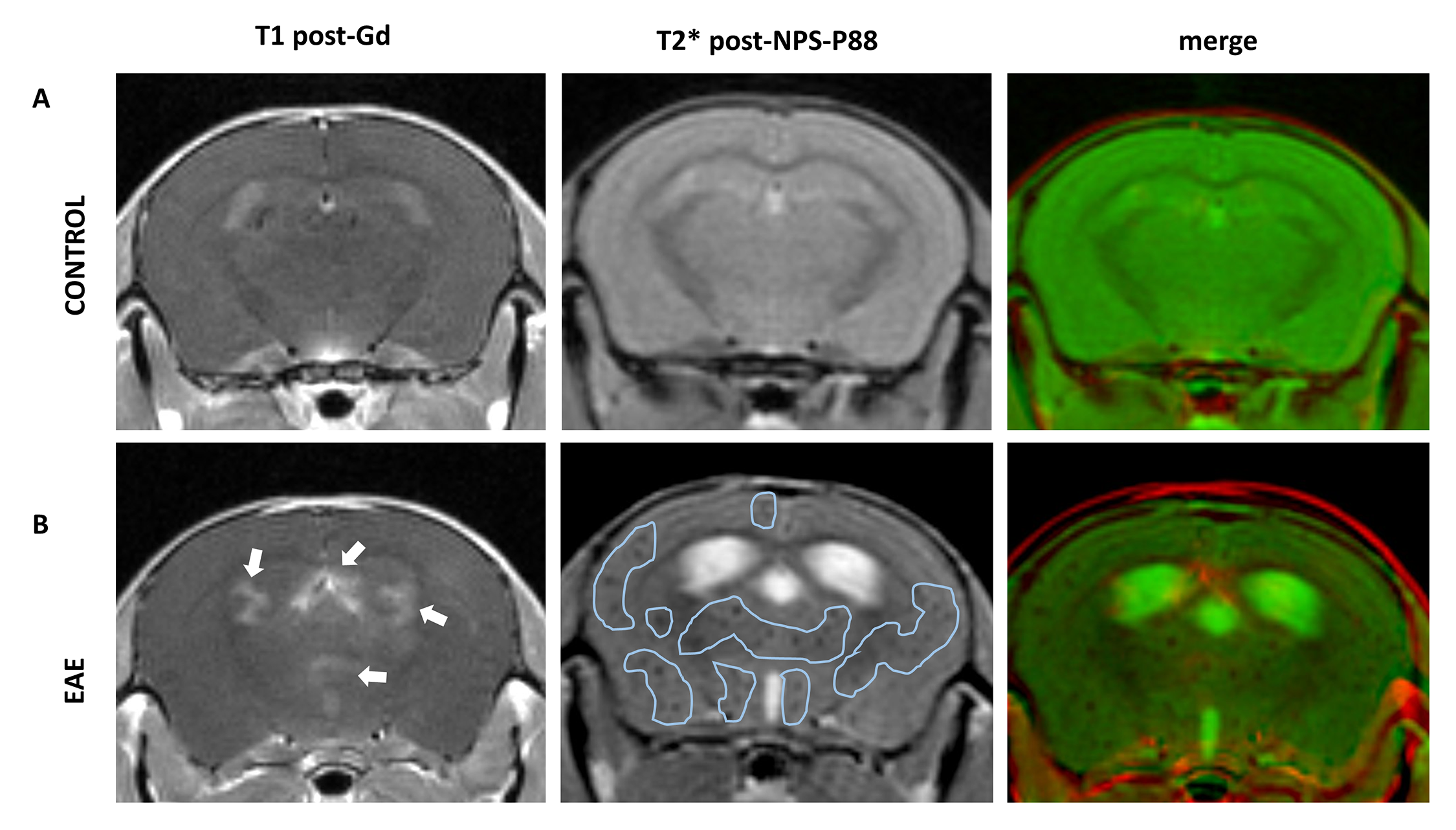

Supplement: S9 Fig — A) MRI image in control animal post to Gd injection, NPS-P88 injection and Merge. B) MRI image in EAE animal after Gd injection, NPS-P88 and Merge (in red T1 for Gd and green T2* for NPS-P88). (TIF) [file pone.0302031.s009.tif]

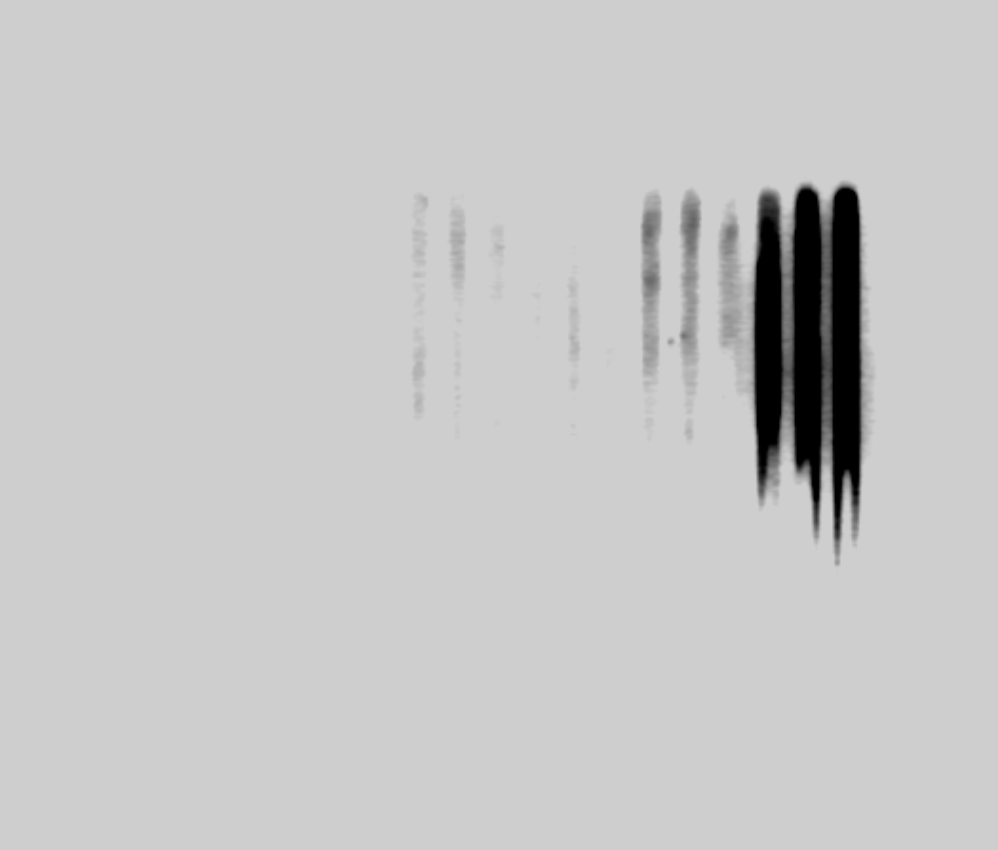

Supplement: S1 Raw images — The image presented in Fig 2 is a cropped version of this image, modified to improve the presentation of the results after removing irrelevant lanes and including the labels for each of the columns. (TIF) [file pone.0302031.s010.tif]
